# Enhancing CAR‐T Cell Efficacy in Solid Tumors by Inhibiting CCL5/VEGF‐Mediated Angiogenesis

**DOI:** 10.1002/advs.202521975

**Published:** 2026-05-06

**Authors:** Shishuo Sun, Qihong Li, Bixi Wang, Gang Wang, Ying Xue, Yibing Liang, Chenhe Hu, Zhenyu Wang, Xiaoge Gao, Haiheng Xu, Jun Song, Hailong Li, Liantao Li, Xiaoxiao Liu, Patrick Ming‐Kuen Tang, Qing Zhang

**Affiliations:** ^1^ Cancer Institute Cellular Therapeutics School of Medicine Xuzhou Medical University Xuzhou Jiangsu P.R. China; ^2^ Center of Clinical Oncology The Affiliated Hospital of Xuzhou Medical University Xuzhou Jiangsu P.R. China; ^3^ Jiangsu Center for the Collaboration and Innovation of Cancer Biotherapy Xuzhou Medical University Xuzhou Jiangsu P.R. China; ^4^ Department of General Surgery The Affiliated Hospital of Xuzhou Medical University Xuzhou Jiangsu China; ^5^ Department of Urology The Affiliated Hospital of Xuzhou Medical University Xuzhou Jiangsu P.R. China; ^6^ Department of Radiation Oncology Cancer Center The Affiliated Hospital of Xuzhou Medical University Xuzhou Jiangsu P.R. China; ^7^ Department of Anatomical and Cellular Pathology State Key Laboratory of Translational Oncology The Chinese University of Hong Kong Shatin Hong Kong P.R. China

**Keywords:** CAR‐T, CCL5, solid tumor, tumor angiogenesis, VEGF

## Abstract

Chimeric antigen receptor‐modified T cells (CAR‐T) have shown remarkable success in hematologic malignancies, but their efficacy against solid tumors remains limited. While immunosuppressive cells and molecules in the tumor microenvironment (TME) are known to impair CAR‐T function, these are not CAR‐T‐specific barriers. Using multiple mouse models, we found that the impact of CAR‐T cells on tumor growth is dose‐dependent, capable of promoting, having no effect on, or inhibiting tumor growth. Mechanistically, tumor‐infiltrating CAR‐T cells play a dual role: they release antitumor effector molecules (e.g., IFN‐γ, TNF‐α), but also produce CCL5, which promotes tumor growth by inducing VEGF and angiogenesis. CCL5‐mediated protumor activity was identified as a key limiting factor for CAR‐T efficacy. Importantly, combining CCL5‐knockout CAR‐T cells with the CCR5 inhibitor maraviroc significantly enhanced antitumor efficacy. These findings reveal a mechanism constraining CAR‐T function in solid tumors and suggest promising combination strategies to improve therapeutic outcomes.

## Graphical abstract

This study reveals that CAR‐T cells in solid tumors produce CCL5, which paradoxically induces VEGF and angiogenesis to promote tumor growth. Blocking CCL5/VEGF signaling—through gene knockout, or the CCR5 inhibitor maraviroc—significantly enhances the antitumor efficacy of CAR‑T therapy (the diagram was created in *Biorender*).

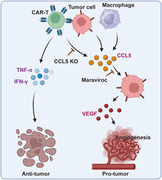

**Abbreviations**
ACT: adoptive cell therapy
CAIX: carbonic anhydrase IX
CAR‐T: chimeric antigen receptor‐modified T
Cy: Cyclophosphamide
DC: dendritic cell
DMEM: Dulbecco's Modified Eagle Medium
E:T: effector cell: target cancer cell
ECM: extracellular matrix
ELISA: Enzyme‐linked immunosorbent assay
EMT: epithelial‐mesenchymal transition
EpCAM: epithelial cell adhesion molecule
FACS: Flow cytometry
GSEA: gene set enrichment analysis
IFN‐γ: Interferon‐gamma
IL‐2: interleukin‐2
i.p.: intraperitoneal injection
i.v.: intravenous injection
PBMC: peripheral blood mononuclear cells
Q‐PCR: real‐time quantitative PCR
rhCAIX: recombinant human CAIX
rhEpCAM: recombinant human EpCAM
RTCA: Real‐time cellular analysis
s.c.: subcutaneous transplantation tumor
Treg: regulatory T cell
TAM: tumor‐associated macrophages
TGR: tumor growth rate
TME: tumor microenvironment
TNF‐α: Tumor necrosis factor‐α
VEGF: vascular endothelial growth factor

## 1 Introduction

Chimeric antigen receptor‐modified T cell (CAR‐T) therapy has demonstrated remarkable success in addressing hematological malignancies, with complete response rates exceeding 90% for acute lymphoblastic leukemia and over 60% for non‐Hodgkin's lymphoma [1, 2]. To date, a total of thirteen CAR‐T cell products targeting 12 distinct indications of hematological cancers have received approval in both the United States and China [3].

The clinical success of CAR‐T therapy in hematologic malignancies has generated great expectations for its performance in solid tumors. Recent clinical studies have shown that CAR‐T therapy targeting GD2 for neuroblastoma [4, 5], targeting IL13Rα2 for glioblastoma [6], targeting GPC3 for hepatocellular carcinoma [7], targeting CEA for liver metastases [8, 9], and targeting CLDN18.2 for gastrointestinal tumors [10] demonstrate certain efficacy. However, these responses pale in comparison to the overwhelming success seen in hematologic malignancies, and most studies are single‐center trials with limited sample sizes.

The stark difference in efficacy between hematologic and solid tumors can be attributed to multiple factors, including the heterogeneity or even complete absence of tumor antigens, the physical and immune barriers within tumors, and the immunosuppressive tumor microenvironment [11]. The immunosuppressive effects of the tumor microenvironment (TME) are one of the main factors limiting the efficacy of CAR‐T therapy in solid tumors [12, 13]. Previous studies have suggested that immune‐suppressive cells in the tumor microenvironment (such as Tregs and TAMs), immune‐suppressive molecules (such as PD‐L1) are important factors limiting the efficacy of CAR‐T therapy in solid tumors [14, 15]. In fact, these immune‐suppressive factors apply to all immunotherapeutics and are not specific to CAR‐T therapy. Researchers have made a series of modifications to CAR‐T cells based on these factors or developed combination treatment strategies, yet a definitive breakthrough in solid tumor treatment still remains to be achieved [16]. Therefore, there is a need to continue investigating the underlying mechanisms for the poor efficacy of CAR‐T therapy in solid tumors, with the aim of developing new CAR‐T technologies or combination treatment strategies.

In this study, we found that during CAR‐T therapy for tumors, on one hand, the cytokines released by CAR‐T cells, such as IFN‐γ, and TNF‐α, etc., exhibit anti‐tumor effects; on the other hand, CCL5 produced by CAR‐T cells, etc., promotes tumor growth by inducing VEGF expression and angiogenesis. The pro‐tumor effect mediated by CCL5 is an important factor limiting the efficacy of CAR‐T therapy, and intervening in the CCL5 signaling pathway can significantly enhance the therapeutic effect of CAR‐T on solid tumors. These findings will further elucidate the underlying mechanism for the poor efficacy of clinical CAR‐T therapy in solid tumors and provide a basis for the development of new CAR‐T treatment technologies and combination treatment strategies.

## 2 Results

### 2.1 CAR‐T Therapy in Solid Tumor Shows a Paradoxical Dose‐Dependent Effect

**FIGURE 1:**
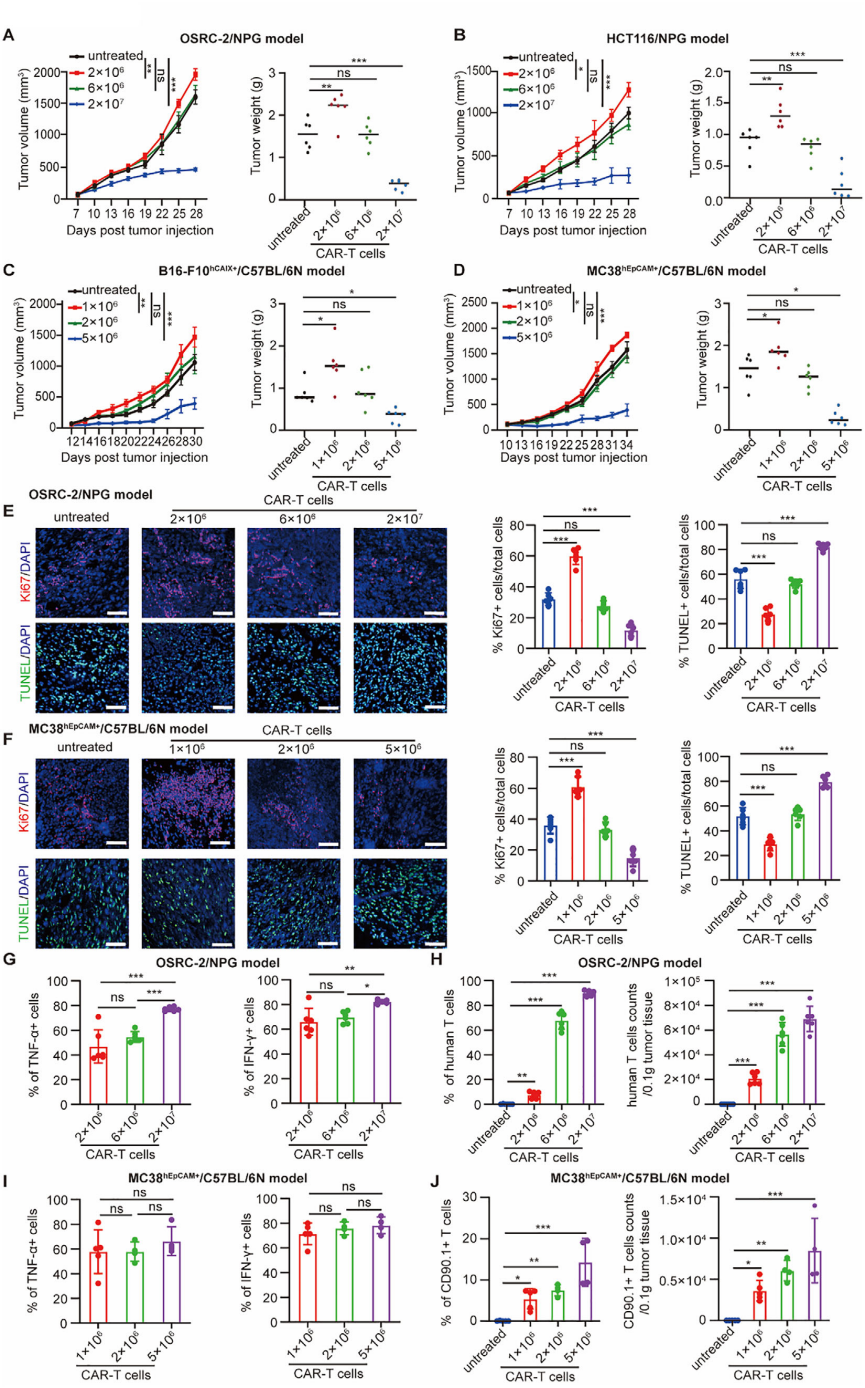
Biphasic impact of CAR‐T cell dose on solid tumor progression. (A) Therapeutic efficacy of human CAIX‐CAR‐T cells against human renal cancer xenografts in immunodeficient (NPG) mice. Mice bearing OSRC‐2 subcutaneous tumors were treated with varying doses of CAR‐T cells (2 × 10^6^, 6 × 10^6^, or 2 × 10^7^) or left untreated on day 7 (*n* = 6). Tumor growth kinetics and final tumor weights were recorded. (**B)** Therapeutic efficacy of human EpCAM‐CAR‐T cells against human colon cancer xenografts in NPG mice. HCT116 tumor‐bearing mice were treated with CAR‐T cells at the same dose range or left untreated (*n* = 6). Tumor growth and endpoint weights were presented. (C) Biphasic impact on therapeutic efficacy of mouse CAIX‐CAR‐T to melanoma model in C57BL/6N mice. C57BL/6N mice were subcutaneously injected with B16‐F10^hCAIX+^ cells (2 × 10^5^), preconditioned with Cyclophosphamide (Cy) on day 11, and treated on day 12 with CAIX‐CAR‐T cells (1 × 10^6^, 2 × 10^6^, or 5 × 10^6^) or left untreated (*n* = 6). Tumor growth and final weights were recorded. (D) Biphasic impact on therapeutic efficacy of mouse EpCAM‐CAR‐T to colon cancer model in C57BL/6N mice. C57BL/6N mice were injected with MC38^hEpCAM+^ cells (1 × 10^6^), preconditioned with Cy on day 9, and treated on day 10 with Thy‐1.1+murine‐derived EpCAM‐CAR‐T cells (1 × 10^6^, 2 × 10^6^, or 5 × 10^6^) or left untreated (*n* = 6). Tumor growth and endpoint weights were presented. The mouse body weight data for (A–D) were shown in Figure S2E–H. (E) Immunohistochemical analysis of OSRC‐2 tumor xenografts. After sacrifice, tumor tissues from the OSRC‐2 model were harvested, fixed in formaldehyde, paraffin‐embedded, sectioned, and subjected to fluorescent staining for Ki67 and TUNEL. Images were acquired by microscopy. Fluorescence intensity was quantified using ImageJ, and the positive rates for Ki67 and TUNEL were statistically analyzed, Bar indicated 50 µm (*n* = 6). (F) Immunohistochemical analysis of MC38^hEpCAM+^ tumors. After sacrifice, tumor tissues from the MC38^hEpCAM+^ model were harvested, fixed in formaldehyde, paraffin‐embedded, sectioned, and subjected to fluorescent staining for Ki67 and TUNEL. Images were acquired by microscopy. Fluorescence intensity was quantified using ImageJ, and the positive rates for Ki67 and TUNEL were statistically analyzed, Bar indicated 50 µm (*n* = 6). (G) Cytokine production by tumor‐infiltrating CAR‐T (human CD45+, human CD3+) cells in OSRC‐2 tumors. At the experimental endpoint, single‐cell suspensions were prepared from OSRC‐2 tumor tissues. After 6‐h stimulation with PMA, ionomycin, and brefeldin A (BFA), the capacity of tumor‐infiltrating CAR‐T cells to produce TNF‐α and IFN‐γ was analyzed by flow cytometry (*n* = 6). (H) Quantification of tumor‐infiltrating CAR‐T cells in OSRC‐2 tumors. At the experimental endpoint, single‐cell suspensions were prepared from OSRC‐2 tumor tissues. The proportion and absolute number of tumor‐infiltrating CAR‐T cells (human CD45^+^, human CD3^+^) were analyzed by flow cytometry (*n* = 6). Gating strategy of (G, H) was shown in Figure S5A–C. (I) Cytokine production by tumor‐infiltrating CAR‐T cells in MC38^hEpCAM+^ tumors. At the experimental endpoint, single‐cell suspensions were prepared from MC38^hEpCAM+^ tumor tissues. After 6‐h stimulation with PMA, ionomycin, and BFA, the capacity of tumor‐infiltrating CAR‐T cells to produce TNF‐α and IFN‐γ was analyzed by flow cytometry (*n* = 6, except in cases where tumor was too small for data collection). (J) Quantification of tumor‐infiltrating CAR‐T (CD90.1+) cells in MC38^hEpCAM+^ tumors. At the experimental endpoint, single‐cell suspensions were prepared from MC38^hEpCAM+^ tumor tissues. The proportion and absolute number of tumor‐infiltrating CAR‐T cells (murine CD90.1+) were analyzed by flow cytometry (*n* = 6, except in cases where tumor was too small for data collection). Gating strategy of (I, J) was shown in Figure S5D–F. Statistical differences between tumor growth curves (A–D) were analyzed by two‐way repeated‐measures ANOVA followed by Dunnett's multiple‐comparisons test. Other data were represented as mean ± SD and were analyzed using one‐way ANOVA. ^*^ *p* < 0.05; ^**^ *p* < 0.01; ^***^ *p* < 0.001; ns, no significance for indicated comparison.

To evaluate the dose‐response relationship of CAR‐T cell therapy in solid tumors, we developed human‐derived second‐generation CAR‐T cells with 4‐1BB costimulatory domain (CSD), targeting tumor‐associated antigen carbonic anhydrase IX (CAIX) for renal carcinoma or epithelial cell adhesion molecule (EpCAM) for colorectal cancer (Figure S1). Subcutaneous xenograft models were established in NPG mice (NOD‐*Prkdc^scid^Il2rg^null^*) using human renal carcinoma OSRC‐2 cells and human colon carcinoma HCT116 cells. Seven days post‐tumor implantation, three dose cohorts (2 × 10^6^, 6 × 10^6^, or 2 × 10^7^ cells/mouse) of CAR‐T cells targeting CAIX or EpCAM were administered via tail vein injection (i.v.) (Figure S2A,B). Notably, we observed a paradoxical dose‐dependent effect. The administration of 2 × 10^7^ CAR‐T cells resulted in significant tumor suppression, as evidenced by attenuated tumor growth kinetics and reduced terminal tumor weights. In contrast, the 6 × 10^6^ cell dose exhibited no statistically significant therapeutic effect. Strikingly, the 2 × 10^6^ cell dose paradoxically enhanced tumor progression, leading to accelerated tumor volume expansion and increased final tumor mass compared to untreated controls (Figure 1A,B). These findings demonstrate that the efficacy of human CAR‐T cell therapy shows a paradoxical dose‐dependent effect in immunodeficient mouse models with human xenograft.

To further verify the dose‐dependent therapeutic effects of CAR‐T cells on solid tumors in immunocompetent wild‐type murine models, we established human CAIX‐overexpressing mouse melanoma B16‐F10 cell line (B16‐F10^hCAIX+^) and human EpCAM‐overexpressing mouse colon cancer MC38 cell line (MC38^hEpCAM+^) (Figure S3), and use the cell lines to establish subcutaneous melanoma or colon tumor models in immunocompetent C57BL/6N mice, respectively. Thy‐1.1+ (CD90.1+) mouse‐derived CAR‐T cells targeting hCAIX or hEpCAM were developed (Figure S4), and doses of 1 × 10^6^, 2 × 10^6^, and 5 × 10^6^ CAR‐T cells were administered via tail vein injection (i.v.) (Figure S2C,D). The 5 × 10^6^ dose significantly inhibited tumor growth, while the 2 × 10^6^ dose had no significant impact. Surprisingly, the 1 × 10^6^ dose promoted tumor growth (Figure 1C,D). Throughout the treatment period, no significant differences in mouse body weight were observed among the groups (Figure S2E–H), indicating that these paradoxical effects were not attributable to systemic toxicity or overall health deterioration. These data further demonstrate the nonlinear dose–response relationship of CAR‐T therapy against solid tumors in immunocompetent wild‐type murine models.

To assess the functional impact of CAR‐T cells, tumor tissues harvested after administration of different CAR‐T cell doses were evaluated for proliferation (Ki67) and apoptosis (TUNEL) using immunofluorescence staining. In the immunodeficient OSRC‐2 model, treatment with 2 × 10^6^ CAR‐T cells increased the number of Ki67^+^ cells and reduced apoptotic rates, while higher doses progressively suppressed Ki67 expression and enhanced apoptosis (Figure 1E). This observation is consistent with the paradoxical dose‐dependent effect of CAR‐T cells in solid tumors. A similar trend was observed in immunocompetent mice bearing MC38^hEpCAM+^ tumors: initial CAR‐T cell administration elevated Ki67 levels and decreased apoptosis, whereas dose escalation led to reduced Ki67 expression and increased apoptotic activity (Figure 1F). These data imply that inappropriate low‐dose CAR‐T cell therapy may promote tumor growth through unknown mechanisms.

In parallel, cytokine profiling of tumor‐infiltrating CAR‐T cells was performed via flow cytometry, based on prior evidence implicating TNF‐alpha (TNF‐α) and interferon‐gamma (IFN‐γ) in CAR‐T–mediated antitumor responses [17, 18]. Notably, CAR expression undergoes internalization and degradation upon antigen stimulation [19], which precludes the use of CAR detection to accurately reflect the level of infiltrating CAR‐T cells in tumors. Therefore, it is common practice to assess adoptively transferred CAR‐T cells by detecting human CD3 [20]. Accordingly, in the immunodeficient OSRC‐2 tumor model, we used human CD45 and CD3 as a surrogate to evaluate CAR‐T cells (Figure S5A–C). Under this approach, the proportion of TNF‐α+ and IFN‐γ+ CAR‐T (human CD45+, CD3+) cells increases with escalating CAR‐T doses, correlating with enhanced antitumor activity (Figure 1G). As anticipated, greater CAR‑T cell infiltration was observed with higher infusion doses (Figure 1H). In immunocompetent mice, CAR‐T cell infiltration in tumors was assessed by detecting CD90.1+ (Figure S5D–F). In the MC38^hEpCAM+^ tumors model, the proportion of TNF‐α^+^ or IFN‐γ^+^ CAR‐T cells (CD90.1+, T cells) remained stable, yet total tumor‐infiltrating CAR‐T cell numbers increased with higher infusion doses (Figure 1I,J). This indicates that greater CAR‐T cell administration expands the absolute number of cytokine‐secreting cells, thereby sustaining antitumor efficacy even without an increase in the relative frequency of cytokine‐producing cells. These results demonstrate that escalating CAR‐T cell doses enhances the absolute quantity of cytokine‐competent CAR‐T cells within tumors, ultimately contributing to effective antitumor responses.

### 2.2 Upregulation of CCL5 in Tumor Tissues Induced by CAR‐T Therapy Plays a Pro‐Tumorigenic Role

The varying outcomes of CAR‐T therapy, ranging from “tumor growth suppression” to “inefficacy” and even “paradoxical tumor promotion”, raise an intriguing question. Based on these dose‐dependent divergent effects, we reasoned that during CAR‐T treatment, cytokines such as TNF‐α, and IFN‐γ, etc., released by CAR‐T cells exert “anti‐tumor effects” [17]. while, certain cytokines or chemokines generated during CAR‐T therapy may mediate “pro‐tumor effects”. We therefore postulated that at lower doses of CAR‐T therapy, the pro‐tumor effects may outweigh the anti‐tumor effects, leading to tumor progression; at higher doses, the anti‐tumor effects prevail, resulting in tumor suppression; and at intermediate doses, these opposing effects may balance each other out, leading to treatment inefficacy. Thus, the pro‐tumor cytokines and chemokines produced during CAR‐T therapy could be critical determinants limiting therapeutic efficacy.

**FIGURE 2:**
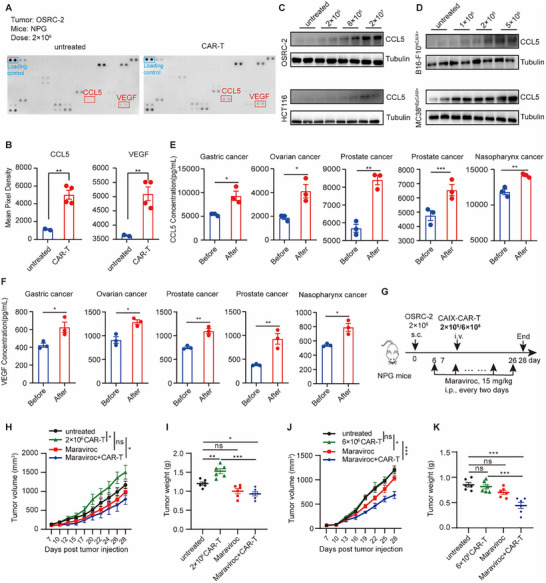
Upregulation of CCL5 in tumor tissues induced by CAR‐T therapy plays a pro‐tumorigenic role. (A) Cytokine array profiling of OSRC‐2 tumor tissues from NPG mice 3 weeks post‐treatment with CAIX‐CAR‐T cells (2 × 10^6^) vs. untreated controls. (B) Quantification of CCL5 and VEGF levels from (A) (untreated, *n* = 2; CAR‐T, *n* = 4). (C,D) Immunoblot analysis of CCL5 in OSRC‐2/HCT116 (C) and B16‐F10^hCAIX+^/MC38^hEpCAM+^ (D) tumors post‐CAR‐T therapy as described in Figure 1A–D (*n* = 6, blots shown are two representative results). (E,F) Plasma CCL5 (E) and VEGF (F) levels measured by ELISA in solid tumor patients receiving CAR‐T treatment (*n* = 5). Serum samples were collected before CAR‐T cell infusion (Before) and 24 h post‐infusion (After). (G) Schematic diagram of the experiment design: NPG mice bearing OSRC‐2 xenografts (2 × 10^6^ cells) received Maraviroc (15 mg/kg) from day 6 followed by CAIX‐CAR‐T cells (2 × 10^6^ or 6 × 10^6^; *n* = 6). i.p, intraperitoneal injection. (H,I) Inhibiting the CCL5/CCR5 signaling pathway significantly abrogates the tumor‐promoting effect of low‐dose (2 × 10^6^) CAR‐T cells against OSRC‐2 tumors in NPG mice. Tumor growth were monitored for over 3 weeks (H). Final tumor weights at endpoint (I). (J,K) Inhibiting the CCL5/CCR5 signaling pathway significantly enhances the therapeutic efficacy of mid‐dose (6 × 10^6^) CAR‐T cells against OSRC‐2 tumors in NPG mice. Tumor growth were monitored for over 3 weeks (J). Final tumor weights at endpoint (K). The mouse body weight data for (G) were shown in Figure S7A,D. Data (B,E,F) were represented as mean ± SD and were analyzed using the *t*‐test. Data (I,K) were represented as mean ± SD and were analyzed using one‐way ANOVA. Statistical differences between tumor growth curves (H,J) were analyzed by two‐way repeated‐measures ANOVA followed by Dunnett's multiple‐comparisons test. ^*^ *p* < 0.05; ^**^ *p* < 0.01; ^***^ *p* < 0.001; ns, no significance for indicated comparison.

To validate this hypothesis and identify the “pro‐tumor” cytokines or chemokines generated during CAR‐T therapy, we collected tumor tissues from both the untreated group and the 2 × 10^6^ cells/mouse CAR‐T treatment group in the CAIX‐specific CAR‐T‐treated NPG mouse model of human renal cell carcinoma OSRC‐2 cell line. Total proteins were extracted, and human cytokine expression profiles were analyzed using a Cytokine Array assay (R&D systems, ARY022B). The results indicated that, compared to the untreated group, the expression levels of CCL5 and vascular endothelial growth factor (VEGF) in the tumor tissues of the CAR‐T treatment group were significantly upregulated, with CCL5 exhibiting the most pronounced increase (Figure 2A,B). To further confirm these findings, we measured CCL5 protein in tumor tissues from the four CAR‐T‐treated tumor models using Western blot. The results revealed low CCL5 expression in the untreated group and significantly higher expression in the CAR‐T treatment group. Moreover, CCL5 expression displayed a dose‐dependent pattern and was positively correlated with the therapeutic dosage of CAR‐T cells (Figure 2C,D). These findings demonstrate that CAR‐T therapy for solid tumors induces the upregulation of CCL5 expression in tumor tissues.

To explore the clinical relevance of these observations, we collected blood samples from patients who received B7H3‐, or gp350‐specific CAR‐T therapy at the Affiliated Hospital of Xuzhou Medical University, including one patient with gastric cancer, one with ovarian cancer, two with prostate cancer, and one with nasopharynx cancer. Blood samples were collected before CAR‐T cell infusion and 24 h post‐infusion. All patients developed fever post‑infusion, indicating in vivo CAR‑T cell activation. ELISA analysis revealed that the plasma concentration of CCL5 and VEGF significantly increased after CAR‐T therapy in all patients (Figure 2E,F). Despite the limited sample size, these results suggest that clinical CAR‐T treatment for solid tumors may also upregulate CCL5 and VEGF expression.

Recent studies have highlighted the dual role of CCL5 in cancer: on one hand, it recruits T cells and dendritic cells (DCs) into tumors, promoting anti‐tumor immunity in certain malignancies [21, 22, 23, 24, 25]; on the other hand, it facilitates tumor progression through various mechanisms: 1) inducing tumor cells, cancer stem cells, and endothelial cells to secrete VEGF, thus promoting tumor angiogenesis; 2) enhancing cancer cell migration, invasion, epithelial‐mesenchymal transition (EMT), and cancer stem cell self‐renewal; 3) recruiting macrophages and inducing their polarization toward the immunosuppressive M2 phenotype, thereby driving tumor malignancy; and 4) recruiting regulatory T cells (Tregs) and inducing CD8^+^ T‐cell exhaustion [26]. However, the role of CCL5 in CAR‐T cell therapy for tumors remains unclear.

To investigate the role of CCL5 in the anti‐solid tumor efficacy of CAR‐T cells, we utilized the CCR5 inhibitor, Maraviroc, to block the CCL5/CCR5 signaling pathway in NPG mice bearing the OSRC‐2 tumor model during CAIX‐specific CAR‐T treatment. CCR5, the primary receptor for CCL5 in cancer progression [26], was confirmed to be expressed in the relevant tumor cell lines by immunoblotting (Figure S6). Consistent with prior observations, CAR‑T monotherapy at a dose of 2 × 10^6^ cells promoted tumor growth, an effect that was abrogated by Maraviroc co‑administration (Figure 2G–I). When CAR‑T cells were administered at a dose of 6 × 10^6^ cells, monotherapy exhibited limited efficacy; however, combining this dosage with Maraviroc significantly enhanced antitumor activity (Figure 2G,J,K).

Given that CCL5 is strongly associated with CD8^+^ T‑cell infiltration in patients and activated T cells express CCR5 [21, 27], we assessed the impact of Maraviroc on CAR‑T cell infiltration and effector function in above tumor models. At doses of both 2 × 10^6^ and 6 × 10^6^ CAR‐T infusion, Maraviroc did not significantly alter CAR‑T cell infiltration or TNF‐α expression, but moderately increased IFN‐γ levels (Figure S7B,C,E,F). Furthermore, flow‑sorted tumor‑infiltrating CAR‑T cells from the Maraviroc combined with 6 × 10^6^ CAR‑T treatment group were co‑cultured with OSRC‑2 cells in a real‑time cell analysis (RTCA) assay. Results indicated that Maraviroc combination therapy slightly enhanced the cytotoxic function of these infiltrating CAR‑T cells (Figure S7G). Notably, no significant differences in body weight were observed among the groups during treatment (Figure S7A,D). Collectively, these findings demonstrate that Maraviroc does not adversely affect CAR‑T cells but can directly potentiate their antitumor efficacy. These data indicate that CAR‑T therapy upregulates CCL5 in solid tumors; inhibiting the CCL5/CCR5 axis thereby enhances the therapeutic outcome of CAR‑T treatment.

### 2.3 Upregulation of CCL5 in Tumor Tissues Induced by CAR‐T Therapy Promotes Angiogenesis by Upregulating the Expression of VEGF

**FIGURE 3:**
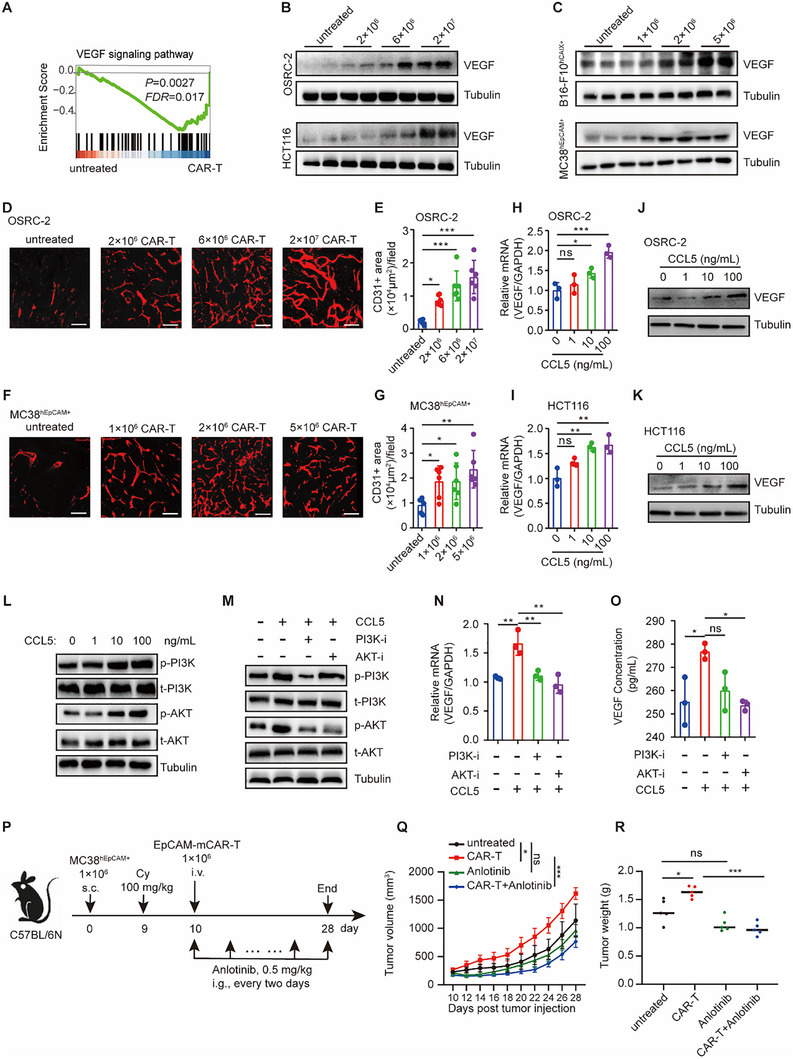
Upregulation of CCL5 in tumor tissues induced by CAR‐T therapy promotes angiogenesis by upregulating the expression of VEGF. (A) Gene Set Enrichment Analysis (GSEA) of VEGF signaling pathway in FACS‐sorted OSRC‐2 tumor cells from NPG mice treated with CAIX‐CAR‐T cells (2 × 10^6^) versus untreated controls. The tumor treatment procedure is provided in Figure S2A. Tumor cells sorted from six mice in untreated or 2 × 10^6^ CAR‐T cell treated group were pooled, and two technical replicates were subjected to RNA sequencing. (B,C) Immunoblot analysis of VEGF expression in OSRC‐2/HCT116 (B) and B16‐F10^hCAIX+^/ MC38^hEpCAM+^ (C) tumors post‐CAR‐T therapy as described in Figure S2A–D, following euthanasia, tumor tissues were collected, and protein extracts were prepared for detection (*n* = 6, blots shown are two representative results). (D–G) Tumor vasculature in OSRC‐2 (D) and MC38 (F) tumor models were detected using whole‐mount immunofluorescence staining. The CAR‐T cell treatment protocol is detailed in Figure 2A,D. After euthanasia, tumor tissues were collected and fixed overnight in 4% paraformaldehyde prior to whole‐mount immunofluorescence staining. Microvessel density quantified by ImageJ (E,G) (*n* = 6 fields/group). Bar indicated 100 µm. (H,I) Q‐PCR detected the VEGF expression in OSRC‐2 (H) and HCT116 (I) cancer cells after CCL5 treatment for 24 h (*n* = 3). (J,K) Dose‐dependent VEGF induction by CCL5 in OSRC‐2 (J) and HCT116 (K) cells were detected using immunoblot. (L) CCL5 induced PI3K/AKT activation in OSRC‐2 cells were detected using immunoblot. (M) Effect of PI3K/AKT inhibitors (30 min pretreatment) on CCL5‐induced PI3K/AKT activation were detected by immunoblot. Experiments (J–M) were performed in triplicate, and similar results were obtained. (N) Q‐PCR analysis of CCL5 mRNA in OSRC‐2 cells pretreated with PI3K/AKT inhibitors (30 min) followed by CCL5 stimulation (24 h) (*n* = 3). (O) ELISA measurement of secreted CCL5 in OSRC‐2 cell supernatants (*n* = 3). (P–R) Inhibiting VEGF‐mediated angiogenesis abrogates the tumor‐promoting effect of low‐dose (1 × 10^6^) CAR‐T cells against MC38^hEpCAM+^ tumors in C57BL/6 mice. Schematic of the experimental design: C57BL/6 mice bearing MC38^hEpCAM+^ xenografts (1 × 10^6^ cells) received anlotinib (0.5 mg/kg) from day 10, followed by administration of EpCAM‐CAR‐T cells (1 × 10^6^; *n* = 5/group). i.g., intragastric administration (P). Tumor growth was monitored for over 3 weeks (Q). Final tumor weights at the experimental endpoint (R). The mouse body weight data for (P) were shown in Figure S9. Statistical differences between tumor growth curves (Q) were analyzed by two‐way repeated‐measures ANOVA followed by Dunnett's multiple‐comparisons test. Other data were represented as mean ± SD and were analyzed using one‐way ANOVA. ^*^ *p* < 0.05; ^**^ *p* < 0.01; ^***^ *p* < 0.001; ns, no significance for indicated comparison.

As previously described, the significant upregulation of CCL5 and VEGF in tumor tissues following CAR‐T treatment was observed (Figure 2A–C). Based on these findings, we hypothesized that CCL5 produced by CAR‐T cells may induce tumor angiogenesis by enhancing VEGF expression, thereby facilitating tumor growth. To test this hypothesis, we sorted tumor cells (CAIX+) from the 2 × 10^6^ cells per mouse CAR‐T treatment group and untreated group in the CAIX‐specific CAR‐T‐treated NPG mouse model of human renal cell carcinoma OSRC‐2 subcutaneous xenografts as described above (Figure S2A). RNA sequencing analysis revealed a substantial upregulation of the VEGF signaling pathway after CAR‐T treatment (Figure 3A). Meanwhile, signaling pathways such as Cell cycle, DNA replication, and Pathways in cancer were also significantly upregulated in the 2 × 10^6^ CAR‐T cell treatment group (Figure S8A), suggesting enhanced tumor progression.

Western blot analysis further confirmed that VEGF expression levels were significantly elevated in the OSRC‐2, HCT116, MC38^hEpCAM+^, and B16‐F10^hCAIX+^ tumor tissues following CAR‐T treatment. Notably, VEGF expression levels exhibited a dose‐dependent relationship with the dosage of CAR‐T treatment (Figure 3B,C). Whole mount immunofluorescence staining experiments demonstrated that CAR‐T treatment increased tumor vascular density; moreover, this increase in vascular density also showed a dose‐dependent relationship with CAR‐T treatment dosage (Figure 3D–G). Additional studies confirmed that recombinant CCL5 protein upregulated VEGF expression in OSRC‐2 and HCT116 cells in vitro (Figure 3H–K). These findings suggest that CCL5 induced by CAR‐T therapy promotes tumor angiogenesis by upregulating VEGF expression.

Previous studies have indicated that CCL5 induces VEGF expression via the PI3K/AKT signaling pathway [28, 29]. To investigate the mechanism through which CCL5 enhances VEGF expression in this study, we assessed the activation of the PI3K/AKT pathway. CCL5 treatment resulted in a dose‐dependent increase in phosphorylated PI3K and AKT levels in OSRC‐2 cells (Figure 3L). Consistently, pharmacological inhibition of the PI3K/AKT signaling pathway using specific antagonists effectively blocked the CCL5‐mediated upregulation of VEGF expression (Figure 3M–O). These data indicate that CCL5 induced by CAR‐T therapy upregulates VEGF expression in tumor tissues via the PI3K/AKT signaling pathway.

To further define the role of VEGF‑mediated angiogenesis in low‑dose CAR‑T‑associated tumor promotion, the anti‑angiogenic multitargeted tyrosine kinase inhibitor, Anlotinib [30], was combined with 1 × 10^6^ CAR‑T cells in immunocompetent MC38^hEpCAM+^‑bearing mice. Anlotinib effectively abrogated the tumor‑promoting effect of low‑dose CAR‑T therapy (Figure 3P–R). Throughout the treatment period, no significant differences in mouse body weight were observed among the groups (Figure S9). These data demonstrate that angiogenesis critically contributes to CAR‑T‑mediated tumor progression in this context.

### 2.4 Increased CCL5 Expression in Tumor Tissue is Primarily Derived from CAR‐T Cells

**FIGURE 4:**
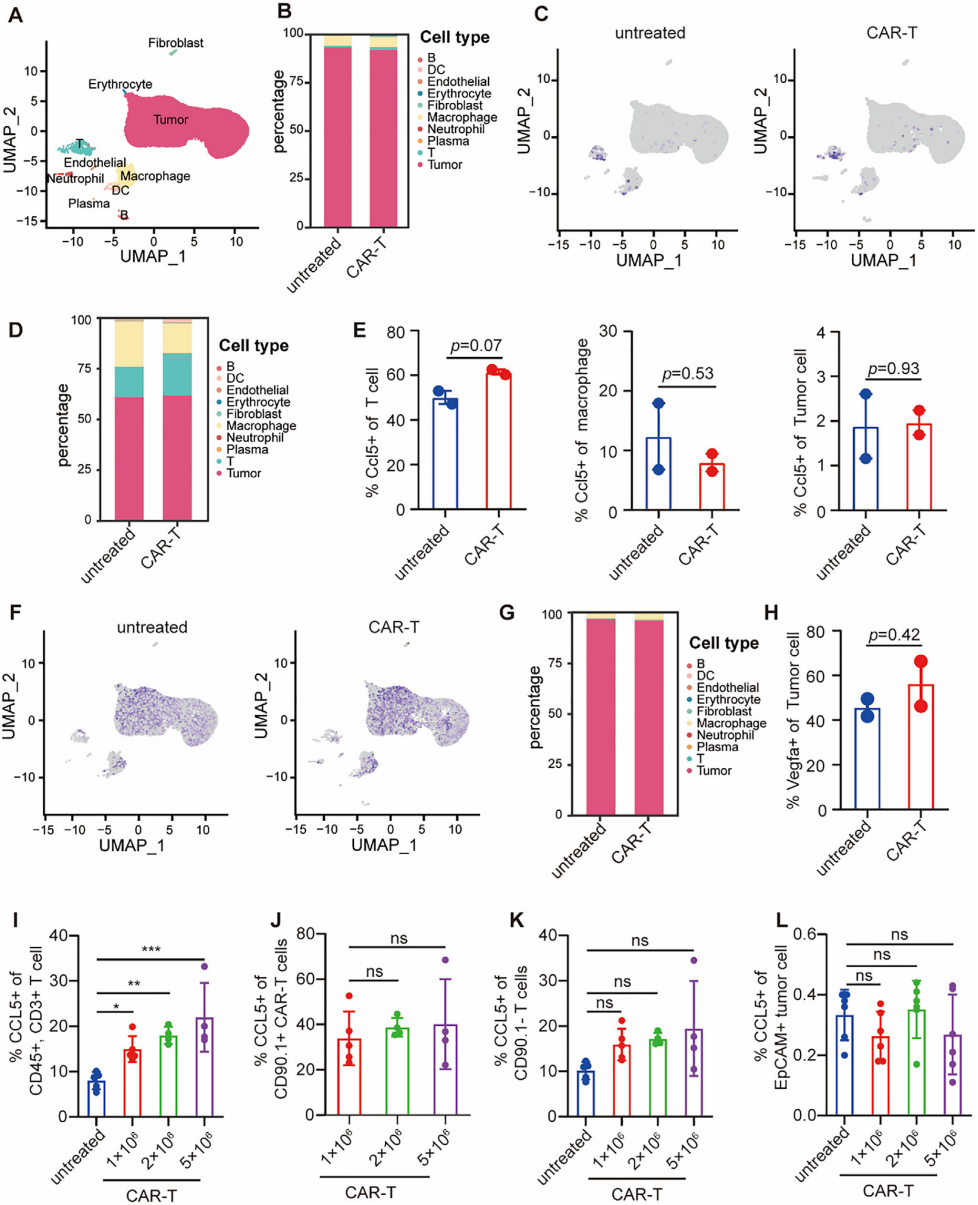
The increased CCL5 expression in tumor tissue is primarily derived from CAR‐T Cells. A‐H. Single‐cell RNA sequencing analysis of MC38^hEpCAM+^ tumors from C57BL/6N mice treated with EpCAM‐CAR‐T cells: Tumor‐bearing mice (1 × 10^6^ MC38^hEpCAM+^ cells) received cyclophosphamide (day 9) followed by CAR‐T cells (day 10), with scRNA‐seq performed at week 4. Two tumor tissues were collected from each group for scRNA‐seq. UMAP plot dataset showing cellular heterogeneity (A). The percentage composition of different cell types (B). UMAP plots highlighting Ccl5 enrichment in untreated group (Left) and CAR‐T treatment group (Right) (C). Proportion of cell subtypes within Ccl5^+^ cells (D). Ccl5 expression frequency in T cells (Left), tumor‐associated macrophages (Middle), and tumor cells (Right) (E). UMAP plots highlighting Vegfa enrichment in untreated group (Left) and CAR‐T treatment group (Right) (F). Proportion of cell subtypes within Vegfa^+^ cells (G). Vegfa expression frequency in tumor cells (H). (I–L) C57BL/6N mice were injected with MC38^hEpCAM+^ cells (1 × 10^6^), preconditioned with cyclophosphamide (Cy) on day 9, and treated on day 10 with Thy‐1.1 murine‐derived EpCAM‐CAR‐T cells (1 × 10^6^, 2 × 10^6^, or 5 × 10^6^), or left untreated (*n* = 6, except in cases where tumor was too small for data collection). FACS analysis of CCL5 expression in tumor‐infiltrating CD3+ T cells (I), CD3+, CD90.1+ CAR‐T cells (J), CD3+, CD90.1‐ endogenous T cells (K), and tumor cells (L). The gating strategies were shown in Figure S10. Data (E,H) were represented as mean ± SD and were analyzed using the *t*‐test. Data (I–L) were represented as mean ± SD and were analyzed using one‐way ANOVA. ^*^ *p* < 0.05; ^***^ *p* < 0.001; ns, no significance for indicated comparison.

To investigate the cellular source of CCL5 in tumors, we established a subcutaneous tumor model using MC38^hEpCAM+^ tumor cells in C57BL/6N mice, which received treatment with hEpCAM‐specific CAR‐T cells derived from T cells of C57BL/6N mice. Four weeks after treatment, the tumor tissues were dissociated into single‐cell suspensions and subjected to single‐cell sequencing analysis. The results showed that CCL5 was primarily expressed in T cells, tumor cells, and macrophages (Figure 4A–D). Further quantification demonstrated that the proportion of CCL5‑expressing T cells increased after CAR‑T therapy, whereas CCL5^+^ tumor cells remained largely unchanged, and CCL5^+^ macrophages decreased (Figure 4E). These results suggest that the upregulation of CCL5 in tumor tissue upon CAR‑T treatment is primarily driven by T cells. In parallel, single‑cell analysis of VEGF expression showed that VEGF was primarily produced by tumor cells, and CAR‑T therapy moderately elevated its expression in this population (Figure 4F–H).

To validate this observation, we generated CAR‑T cells from Thy‐1.1+ murine donor T cells and administered them into tumor‑bearing C57BL/6N mice. Flow cytometry analysis of tumor‑infiltrating cells showed that the frequency of CCL5‑expressing total T cells (CD3+ T cells) rose with increasing CAR‑T doses (Figure 4I, and Figure S10). Further dissection revealed that CAR‑T cells (CD3+, CD90.1+) exhibited a CCL5+ rate of approximately 35%, significantly higher than the baseline ∼10% observed in T cells from untreated tumors. This elevated CCL5 expression in CAR‑T cells remained stable across different treatment doses (Figure 4J). In contrast, non‑CAR‑T cells (CD3+, CD90.1‐) displayed only a marginal increase in CCL5 expression upon CAR‑T therapy (Figure 4K). Meanwhile, tumor cells maintained consistent levels of CCL5 throughout treatment (Figure 4L). Together, these data demonstrate that although CCL5 is expressed by multiple cell types in the tumor microenvironment, the CAR‑T‑induced elevation of CCL5 is predominantly attributable to tumor‑infiltrating CAR‑T cells themselves.

To investigate whether CAR‑T therapy promotes tumor growth by recruiting immunosuppressive cells into the tumor microenvironment, we analyzed single‑cell RNA sequencing data and found no significant differences in the proportions of major immune cell populations—including tumor‑associated macrophages (TAM), DCs, B cells, and neutrophils—between untreated and CAR‑T‑treated tumors (Figure S11A). Flow cytometric analysis further confirmed that the frequencies of classical immunosuppressive subsets, such as TAM and myeloid‑derived suppressor cells (MDSC), remained unchanged in tumors treated with 1 × 10^6^ CAR‑T cells compared with untreated controls (Figure S11B,C). These results exclude the recruitment of heterogeneous immunosuppressive cell subsets as a mechanism underlying the tumor‑promoting effects observed at low CAR‑T cell doses.

### 2.5 Antigen Stimulation Induces CCL5 Expression in CAR‐T Cells via JAK/STAT, NF‐κB, and MAPK Signaling Pathways

**FIGURE 5:**
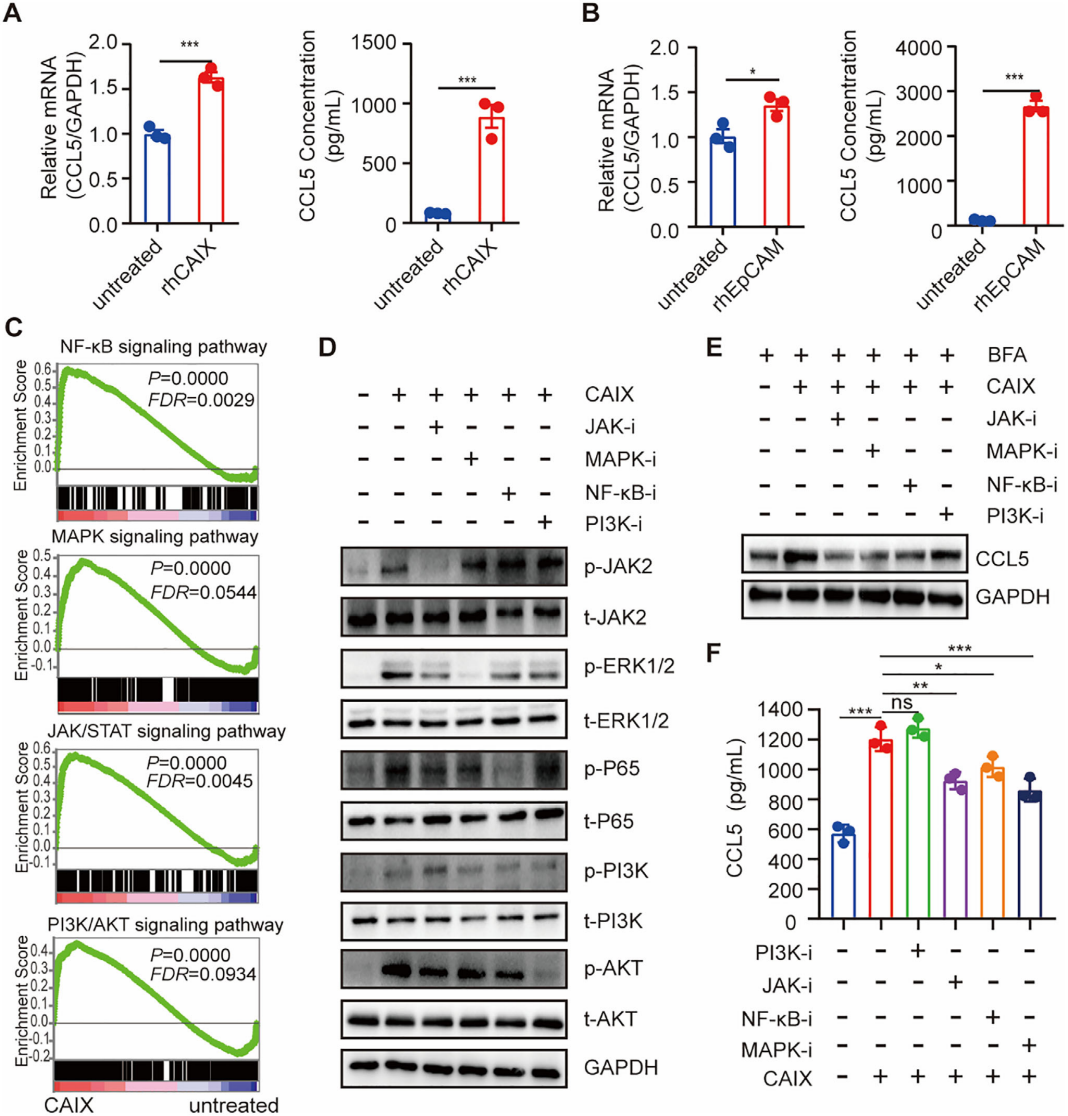
Antigen stimulation induces CCL5 expression in CAR‐T Cells via JAK/STAT, NF‐κB, and MAPK signaling pathways. (A,B) Q‐PCR and ELISA analysis of CCL5 mRNA and protein secretion in CAIX‐CAR‐T (A) and EpCAM‐CAR‐T (B) cells stimulated with recombinant human CAIX (rhCAIX) or EpCAM (rhEpCAM) antigens for 24 h (*n* = 3). (C) GSEA of RNA‐seq data from rhCAIX‐stimulated CAIX‐CAR‐T cells reveals upregulated signaling pathways. CAIX‐CAR‐T cells were stimulated with tumor antigen, rhCAIX, for 24 h in vitro. The stimulation was performed in two separate batches, followed by RNA sequencing. (D) Immunoblot analysis of phosphorylated and total signaling proteins (PI3K, AKT, JAK2, P65, ERK1/2) in CAIX‐CAR‐T cells pretreated with pathway inhibitors (JAK, MAPK, NF‐κB, PI3K) followed by rhCAIX stimulation. (E) Immunoblot analysis of CCL5 protein levels in CAIX‐CAR‐T cells pretreated with pathway inhibitors followed by rhCAIX and Brefeldin A (BFA) stimulation. Experiments (D,E) were performed in triplicate, and similar results were obtained. (F) ELISA quantification of CCL5 secretion in inhibitor‐pretreated CAIX‐CAR‐T cells post rhCAIX stimulation (*n* = 3). Data (A,B) were represented as mean ± SD and were analyzed using the *t*‐test. Data (F) were represented as mean ± SD and were analyzed using one‐way ANOVA. ^*^ *p* < 0.05; ^**^ *p* < 0.01; ^***^ *p* < 0.001; ns, no significance for indicated comparison.

To explore the factors inducing CCL5 secretion by tumor‐infiltrating CAR‐T cells, we hypothesized that tumor antigen stimulation drives CCL5 production in these cells. To confirm this hypothesis, we assessed the mRNA expression and secretion of CCL5 by CAR‐T cells in response to antigen stimulation in vitro. CAIX‐ or EpCAM‐specific CAR‐T cells were stimulated with their respective recombinant antigens, CAIX or EpCAM, and the mRNA expression and protein secretion levels of CCL5 were measured. The results indicated that both mRNA expression and protein secretion levels of CCL5 were significantly elevated in both CAR‐T cell models following stimulation with recombinant antigens (Figure 5A,B).

To further elucidate the mechanisms underlying antigen‐induced CCL5 expression in CAR‐T cells, CAIX‐specific CAR‐T cells underwent RNA sequencing after antigen stimulation. Gene Set Enrichment Analysis (GSEA) revealed that the NF‐κB, MAPK, JAK/STAT, and PI3K/AKT signaling pathways were upregulated in response to CAIX stimulation (Figure 5C). To validate the activation of these signaling pathways upon antigen stimulation, we assessed the phosphorylation of key signaling molecules within these pathways (Figure 5D). The results confirmed their activation following antigen stimulation. We employed inhibitors targeting these signaling pathways to investigate their potential roles in antigen‐induced CCL5 expression. The inhibitors successfully reduced the phosphorylation of the aforementioned signaling molecules, effectively inhibiting their activation (Figure 5D). Notably, inhibition of the JAK/STAT, MAPK, and NF‐κB signaling pathways significantly decreased the expression and secretion of CCL5 by CAR‐T cells in response to antigen stimulation (Figure 5E,F). These findings suggest that antigen stimulation induces CCL5 secretion from CAR‐T cells through the JAK/STAT, MAPK, and NF‐κB signaling pathways.

Given that other components within the complex tumor microenvironment (TME) may influence CCL5 expression by CAR‑T cells, we evaluated the impact of TME‑related factors, including hypoxia, low glucose, and acidic pH, on CAR‑T cells in vitro. Our results indicated that multiple TME conditions can enhance CCL5 secretion by CAR‑T cells (Figure S12A–C). Notably, these effects appeared to be independent of CAR signaling. Consistent with this, approximately 10% of tumor‑infiltrating T cells in untreated mice were CCL5^+^ (Figure 4I), confirming that various TME factors can indeed induce CCL5 expression in infiltrating T cells. A comparison of CCL5 expression in T cells from peripheral blood, spleen, draining lymph nodes (LN), and tumor tissue revealed significantly lower CCL5 levels in non‑tumor sites relative to tumor‑infiltrating T cells (Figure S12D), further supporting the role of the tumor microenvironment in inducing CCL5 expression. However, CAR‑T cells exhibited a significantly higher frequency of CCL5^+^ cells (∼35%) compared to endogenous T cells (∼10%) in the same setting (Figure 4I,J). These findings suggest that while general TME stimuli can promote CCL5 secretion in both endogenous T cells and CAR‑T cells, antigen‑specific stimulation through the CAR is the primary driver of elevated CCL5 production during CAR‑T therapy.

### 2.6 CCL5‐Knockout CAR‐T Cells in Combination with Maraviroc Exhibited Superior Anti‐Tumor Efficacy

Our findings confirm that during CAR‐T cell therapy for solid tumors, CAR‐T cells exhibit dual opposing mechanisms: they inhibit tumor growth by releasing cytotoxic molecules (e.g., TNF‐α) and cytokines (e.g., interferon‐γ), while simultaneously promoting tumor progression through CCL5‐mediated induction of angiogenesis. The ultimate therapeutic outcome depends on the dynamic balance between these opposing forces.

Consequently, the CCL5‐driven pro‐tumorigenic effect represents a major factor limiting CAR‐T efficacy against solid tumors. Notably, CCL5 secretion by CAR‐T cells can be induced upon stimulation by tumor antigens and other factors within the tumor microenvironment (TME) (Figure 5), while, CCL5 is derived not only from CAR‐T cells but also from tumor cells and macrophages (Figure 4D,E). Given these multiple sources of CCL5, we propose that dual targeting of the CCL5/CCR5 axis through “CCL5 knockout in CAR‐T cells” combined with pharmacological CCR5 blockade using the inhibitor maraviroc may substantially enhance the therapeutic efficacy of CAR‐T cell therapy for solid tumors.

**FIGURE 6:**
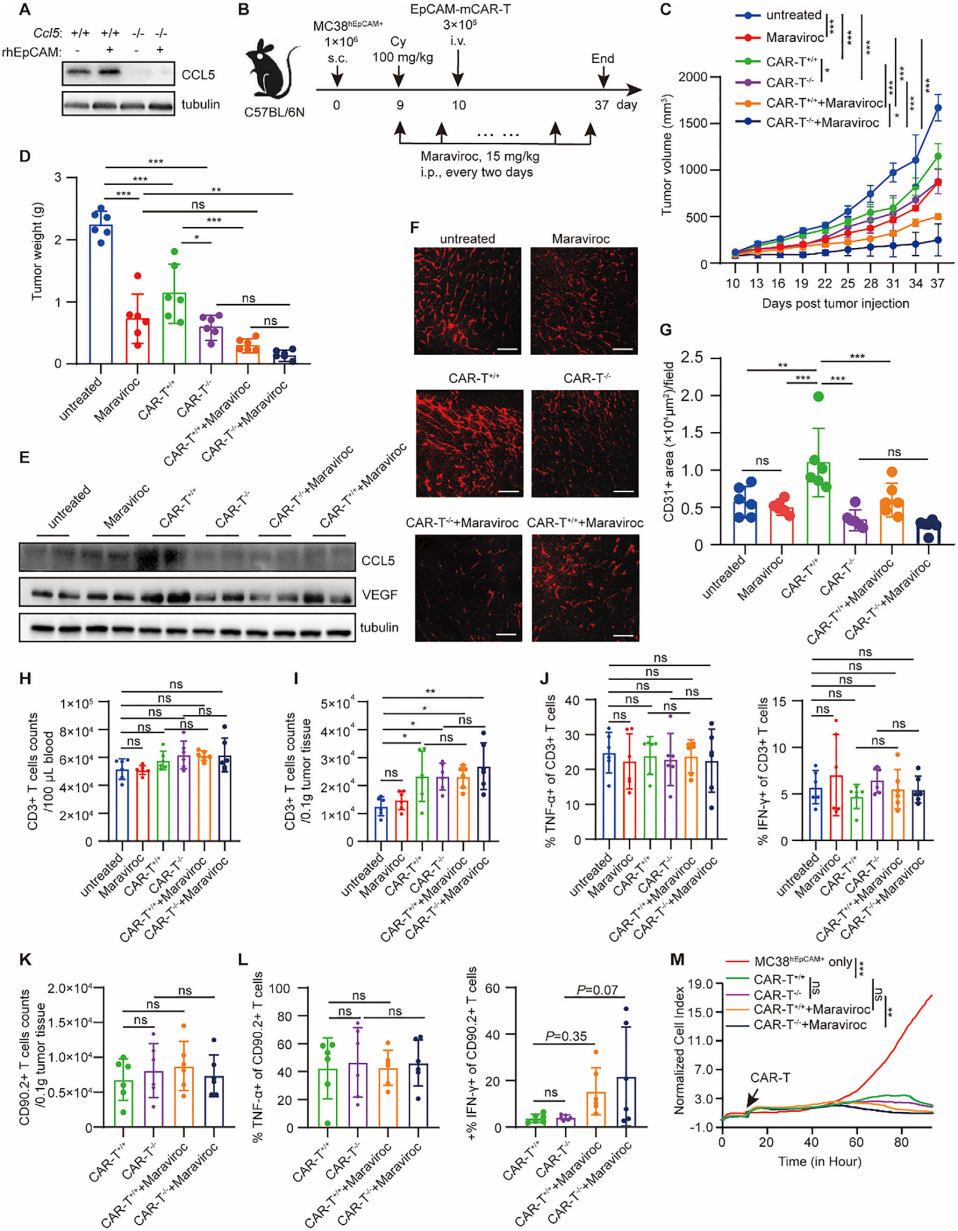
CCL5‐knockout CAR‐T cells combined with maraviroc enhance anti‐tumor efficacy. (A) Western blot validation of CCL5 deficiency in murine EpCAM‐mCAR‐T cells derived from wild‐type (Ccl5+/+) or T cell‐specific CCL5 knockout (Ccl5^−/−)^ Thy‐1.2 C57BL/6N mice, following 24‐h stimulation with rhEpCAM + Brefeldin A. The experiment was performed in triplicate, and similar results were obtained. (B–D) Schematic of the experimental design for combined therapy using CCL5‐deficient CAR‐T cells and Maraviroc in Thy‐1.1 mice with MC38^hEpCAM^ tumor (B). Thy‐1.1 mice were inoculated with 1 × 10^6^ tumor cells, treated with cyclophosphamide (day 9), and randomized to six groups (*n* = 6/group): untreated control, Maraviroc (15 mg/kg), wild‐type CAR‐T (CAR‐T^+/+^), Ccl5^−/−^ CAR‐T (CAR‐T^−/−^), CAR‐T^+/+^ + maraviroc, or CAR‐T^−/−^ + maraviroc. Tumor growth was monitored for 3 weeks (C), and weights were measured at endpoint (D). The mouse body weight data for (B) were shown in Figure S13. (E) Western blot analysis of CCL5 and VEGF expression in tumor tissues (*n* = 6, blots shown are two representative results). (F–G) Whole‐mount immunofluorescence staining of tumor vasculature (F) and quantification of microvessel density using ImageJ (*n* = 6 fields/group) (G). Bar indicated 100 µm. (H) Flow cytometry analysis of peripheral blood T cell, with quantification of absolute T cell count. (I) Flow cytometry analysis of total tumor‐infiltrating T cells (CD3+ T cells), with quantification of absolute T cell count. (J) Flow cytometric analysis of TNF‑α and IFN‑γ expression in total tumor‑infiltrating T cells. (K) Flow cytometric analysis and quantification of tumor‑infiltrating CAR‑T (CD3+, CD90.2+ T cells) cells. (L) Flow cytometric analysis of TNF‑α and IFN‑γ expression in tumor‑infiltrating CAR‑T cells (H–L, *n*=6). Gating strategy of (H–L) was shown in Figure S14. (M) Cytotoxic activity of flow‑sorted tumor‑infiltrating CAR‑T cells co‑cultured with tumor cells at a 2:1 ratio in a RTCA assay. The instrument recorded measurements every 15 min to minimize random error. Statistical differences between tumor growth curves (C) were analyzed by two‐way repeated‐measures ANOVA followed by Dunnett's multiple‐comparisons test. Other data were represented as mean ± SD and were analyzed using one‐way ANOVA. ^*^ *p* < 0.05; ^**^ *p* < 0.01; ^***^ *p* < 0.001; ns, no significance for indicated comparison.

To validate this hypothesis, we generated anti‐hEpCAM CAR‐T cells with CCL5 knockout (CAR‐T^CCL5−/−^) using T cells isolated from T cell‐specific CCL5‐knockout mice (Thy‐1.2+ mice) (Figure 6A). A subcutaneous MC38^hEpCAM+^ colorectal cancer model was established in Thy‐1.1+ mice. On day 9 post‐engraftment, lymphodepletion was performed with cyclophosphamide (Cy). CAR‐T cell therapy (3 × 10^6^ cells/mouse) was administered once on day 10, accompanied by maraviroc treatment every two days. The experiment was terminated on day 37, with peripheral blood and tumor tissues collected for analysis (Figure 6B).

Analysis of endpoint tumor volume and weight revealed that both wild‐type CAR‐T (CAR‐T^+/+^) cells and CCL5 deficiency CAR‐T (CAR‐T^−/−^) cells exhibited anti‐tumor efficacy, though CAR‐T^−/−^ cells demonstrated significantly superior tumor control (tumor growth inhibition, TGI, 73%). While maraviroc monotherapy achieved comparable efficacy to CAR‐T^−/−^ cells, the combination therapy (CAR‐T^−/−^ + maraviroc) resulted in markedly potentiated therapeutic effects, achieving 93% TGI (Figure 6C,D). Importantly, no significant differences in mouse body weight were observed among the groups throughout the treatment period (Figure S13), indicating that the enhanced anti‐tumor efficacy was not attributable to differential systemic toxicity.

Mechanistic analysis revealed that compared to the untreated or maraviroc‐monotherapy group, tumor tissues from CAR‐T^+/+^ group exhibited significantly upregulated CCL5 expression. Conversely, CCL5 levels were substantially reduced in the CAR‐T^−/−^ and combination (CAR‐T^−/−^ + maraviroc) groups. Correspondingly, while VEGF expression was markedly elevated in CAR‐T^+/+^ treated tumors relative to Untreated or maraviroc‐monotherapy group, all therapeutic intervention groups—including CAR‐T^−/−^ monotherapy, CAR‐T^−/−^ plus maraviroc, and CAR‐T^+/+^ plus maraviroc—demonstrated significant downregulation of VEGF (Figure 6E). Whole mount immunofluorescence staining with quantitative analysis confirmed a significant increase in vascular density within tumor tissues from the CAR‐T^+/+^ group compared to Untreated or maraviroc‐monotherapy group. In stark contrast, tumors treated with CAR‐T^−/−^ monotherapy exhibited substantially reduced neovascularization. Most notably, the combination therapy group (CAR‐T^−/−^ + maraviroc) demonstrated the most pronounced reduction in tumor vasculature (Figure 6F,G).

Peripheral blood analysis showed elevated T‐cell counts in all CAR‐T‐treated groups compared to the Untreated or maraviroc‐ monotherapy group, although these differences were not statistically significant (Figure 6H, and Figure S14). In contrast, tumor‐infiltrating T lymphocyte (CD3+ T cells) assessment demonstrated significantly increased T‐cell infiltration in tumor tissues from all CAR‐T therapy groups relative to the Untreated or maraviroc‐monotherapy group. Meanwhile, maraviroc treatment did not alter the tumor‑infiltrating capacity of either total T cells (CD3+ T cells) or CAR‑T cells (CD3+, CD90.2+ T cells) (Figure 6I,K). Meanwhile, maraviroc co‑treatment did not affect TNF‐α or IFN‐γ secretion by total T cells (Figure 6J). However, it moderately enhanced IFN‐γ production specifically by CAR‑T cells (Figure 6L). Furthermore, flow‑sorted tumor‑infiltrating CAR‑T cells analyzed via RTCA exhibited slightly enhanced tumor‑killing activity following maraviroc combination therapy (Figure 6M). These findings collectively demonstrate that CCL5 ablation in CAR‐T cells disrupts the CCL5/CCR5/VEGF pro‐angiogenic axis, and that pharmacological CCR5 blockade synergizes with genetic modification of CAR‐T cells to attenuate the pro‐angiogenic signaling in tumors, thereby significantly enhancing the therapeutic efficacy against solid tumors.

## 3 Discussion

Previous studies have predominantly attributed the limited efficacy of CAR‐T therapy in solid tumors to intrinsic immunosuppressive components of the tumor microenvironment, such as Treg cells, M2 tumor‐associated macrophages, and immune checkpoint molecules like PD‐L1, which are widely recognized as general barriers to immunotherapy rather than CAR‐T‐specific limitations [31]. This study uncovers a dual role of CAR‐T cells in solid tumors: cytokines released by CAR‐T cells, such as TNF‐α and IFN‐γ, exert an antitumor effect, while CCL5 produced by CAR‐T cells, etc., promotes tumor growth by inducing VEGF expression and angiogenesis. The overall therapeutic outcome is influenced by the interplay between these opposing effects as the dose varies. At low doses, the number of effector CAR‐T cells (TNF+, IFN‐γ+) is limited, resulting in insufficient tumor‐killing capacity. Under these conditions, the secreted CCL5 can promote tumor growth. In contrast, at high doses, the number of functional effector CAR‐T cells increases substantially, and their cytotoxic effect becomes dominant, leading to overall tumor inhibition despite the presence of CCL5. This functional paradox underpins the observed dose‐dependent efficacy spectrum, a phenomenon previously unreported in CAR‐T biology. Critically, we demonstrated that genetic ablation of CCL5 in CAR‐T cells combined with pharmacologic CCR5 blockade (maraviroc) disrupted this balance, significantly enhanced therapeutic efficacy against tumors.

In this study, we demonstrate that the pro‑tumor effects observed during CAR‑T therapy are attributed to CCL5 derived from CAR‑T cells, not from tumor cells. Our data indicate that CAR‑T cell therapy leads to a significant increase in CCL5 expression within CAR‑T cells, whereas CCL5 levels in tumor cells remain unchanged. These results confirm that the therapy‑induced elevation of CCL5 originates from CAR‑T cells, not tumor cells. Functionally, CCL5‐knockout CAR‐T cells exhibited superior therapeutic efficacy against tumors compared to wild‐type CAR‐T cells, indicating that CAR‐T cell‐derived CCL5 compromises antitumor effectiveness. Nevertheless, tumor cells exhibit basal CCL5 expression, and consistent with previous reports, this tumor‐derived CCL5 promotes tumor growth [26, 32, 33]. This is further supported by our observation that maraviroc treatment alone, which inhibits the CCL5/CCR5 axis, partially suppresses tumor growth. Thus, while CCL5 from both sources can promote tumor progression, the increase in CCL5 during CAR‑T therapy is specifically derived from CAR‑T cells, excluding the possibility that the therapy‑induced pro‑tumor effect is mediated by tumor‑derived CCL5. The basal expression of CCL5 in tumor cells provides a rationale for combining maraviroc with CAR‑T therapy to block the CCL5/CCR5 pathway.

The discovery of CCL5/VEGF‐mediated angiogenesis as a resistance mechanism aligns with emerging evidence supporting combination strategies targeting tumor vasculature. Previous reports indicate that antiangiogenic agents (e.g., VEGF inhibitors) can normalize tumor vasculature, thereby improving lymphocyte infiltration [34]. Notably, CXCL9‐modified CAR‐T cells have demonstrated dual capabilities in angiogenesis inhibition and enhanced tumor infiltration [35]. In the present study, we evaluated the impact of vascular disruption on CAR‐T therapy using the antiangiogenic inhibitor anlotinib. Our results demonstrate that anlotinib effectively suppresses the tumor‐promoting effect induced by low‐dose CAR‐T treatment. To further elucidate how interfering with VEGF‐driven angiogenesis influences CAR‐T efficacy in solid tumors, the use of VEGF‐knockout tumor cell lines or conditional VEGF‐knockout mouse models would represent a valuable experimental approach. Our findings extend this paradigm by revealing that CCL5 ablation in CAR‐T cells intrinsically disrupts pro‐angiogenic signaling while preserving cytotoxic function. This suggests a triple advantage for combining CCL5‐modulated CAR‐T with antiangiogenic drugs: (1) direct suppression of VEGF‐driven angiogenesis, (2) reduced vascular permeability and interstitial pressure to facilitate CAR‐T trafficking, and (3) mitigation of hypoxia‐induced immunosuppression. Future studies should explore sequential dosing strategies—antiangiogenic pretreatment followed by CCL5‐knockout CAR‐T—to optimize vascular normalization and T‐cell delivery.

Additionally, in a small cohort of five patients treated with B7H3‑ or gp350‑targeted CAR‑T, we observed a post‑treatment rise in plasma CCL5 levels. However, the absence of longitudinal imaging data in these individuals—due to their deteriorating clinical condition and inability to undergo further follow‑up assessments—precludes a direct temporal correlation between CCL5 elevation and radiographic tumor progression. We therefore acknowledge that the association remains observational and hypothesis‑generating rather than causally established. These limitations underscore the necessity for future multi‑center, large‑scale prospective studies that systematically integrate tumor biopsies, serial imaging, and molecular profiling to rigorously evaluate the potential role of CCL5 as a biomarker and its contribution to treatment resistance across diverse solid tumors.

CAR‐T cells interact with the tumor microenvironment in a very complex network of interactions. CAR‐T cells may have multifaceted effects on the tumor microenvironment, including both anti‐tumor effects and pro‐tumor growth effects. Conversely, the tumor microenvironment also has various counteracting effects on CAR‐T cells, including signals that activate CAR‐T cells and signals that inhibit their function [36]. In this study, we identified CCL5, a chemokine that exerts a negative effect during CAR‐T therapy for solid tumors; however, this is just the tip of the iceberg regarding the interactions between CAR‐T cells and the tumor microenvironment. Many other signaling molecules mediating this interaction remain to be uncovered, which will further explore the mechanisms of CAR‐T therapy in solid tumors and enhance the efficacy of CAR‐T treatment in solid tumors.

In conclusion, this study establishes that CAR‐T therapy exerts dual effects in solid tumors, driven by a dynamic balance between cytotoxicity and CCL5‐mediated pro‐angiogenic signaling. The identification of CCL5 as a CAR‐T‐specific resistance factor provides a mechanistic foundation for developing novel strategies, such as CCL5‐knockout CAR‐T cells combined with CCR5 inhibitors such as maraviroc, to disrupt the CCL5/CCR5/VEGF axis and enhance efficacy. While challenges in TME complexity and clinical translation remain, this work redirects focus toward engineering CAR‐T cells with minimized pro‐tumor signaling, offering a promising roadmap for overcoming solid tumor resistance and advancing CAR‐T therapy toward more effective clinical applications.

## 4 Conclusions

In this study, we employed multiple mouse models to demonstrate that CAR‐T cells exhibit a biphasic impact that can either promote tumor growth, show no efficacy, or inhibit tumor growth as the treatment dose increases. Mechanistic studies revealed that tumor‐infiltrating CAR‐T cells play a dual role: effector molecules released by CAR‐T cells, such as granzyme, perforin, and interferon‐γ, exert an antitumor effect, while CCL5 produced by CAR‐T cells, etc., promotes tumor growth by inducing VEGF expression and angiogenesis. The overall therapeutic outcome is influenced by the interplay between these opposing effects as the dose varies. Consequently, the CCL5‐mediated pro‐tumor effect is a critical factor limiting CAR‐T efficacy. Notably, treatment with CCL5‐knockout CAR‐T cells, in combination with the CCL5/CCR5 inhibitor maraviroc, significantly enhanced therapeutic efficacy against tumors. This study elucidates the underlying mechanisms for the limited effectiveness of CAR‐T therapy in solid tumors and has important implications for developing novel CAR‐T treatment strategies and combination therapies aimed at improving CAR‐T efficacy in solid tumors.

## 5 Methods

### 5.1 Materials and Reagents

X‐VIVO medium was purchased from Lonza (Switzerland). RPMI‐1640 and Dulbecco's Modified Eagle Medium (DMEM) medium was obtained from KeyGEN BioTECH (Nanjing, China). Fetal bovine serum (FBS) and Penicillin/streptomycin were purchased from BioChannel Biotechnology Co., Ltd (Nanjing, China). Human T‐Activator CD3/CD28 was obtained from Gibco (11132D), recombinant human IL‐2 (rhIL‐2) was purchased from PrimeGene (Shanghai, China). Maraviroc (CCR5 inhibitor), NSC‐42834 (JAK2 inhibitor) and MK2‐IN‐5 acetate (MAPK inihibitor) were purchased from MedChemExpress (Shanghai, China). SC75741 (NF‐κB inhibitor), LY294002 (PI3K inhibitor) and GSK‐690693 (AKT inhibitor) were purchased from TargetMol (Shanghai, China). Information for the antibodies used for flow cytometry and Western bolt is provided in Tables S1 and S2.

### 5.2 Cell Culture

The human renal cancer cell line OSRC‐2 (Cat. No. 8885651, RRID: CVCL_1626) was purchased from Shanghai Tongwei Biotechnology Co., LTD. on January, 2020. The human colon cancer cell line HCT116 (Cat. No. TCHu 99, RRID: CVCL_0291), the murine melanoma cell line B16‐F10 (Cat. No. SCSP‐5233, RRID: CVCL_0159), and the murine colorectal adenocarcinoma cell line MC38 (Cat. No. SCSP‐5431, RRID: CVCL_B288) were purchased from the National Collection of Authenticated Cell Cultures (Shanghai, China) on September, 2020, May, 2021, and October, 2021, respectively. OSRC‐2, HCT116, B16‐F10 cancer cells were cultured in RPMI‐1640 medium and MC38 cells were cultured in DMEM, respectively. Culture media were supplemented with 10% FBS and 100 unit/mL of penicillin and 100 µg/mL of streptomycin. Cell line authentication and *Mycoplasma* testing were performed by the vendor. B16‐F10^hCAIX+^, MC38^hEpCAM+^ cells were established using B16‐F10 and MC38 cell line by transfecting lentivirus overexpression of human CAIX or EpCAM antigens and selected by puromycin. Human and murine T cells were cultured in X‐VIVO 15 media supplemented with 10% fetal bovine serum, 100 unit/mL of penicillin, 100 µg/mL of streptomycin, and 200 units/mL recombinant interleukin‐2 (IL‐2).

### 5.3 Virus Packaging and CAR‐T Cell Preparation

For the generation of human‐derived CAR‐T cells, second‐generation CAR constructs were placed under the control of the EF1α promoter. Each construct comprised a myc tag, an anti‐human CAIX single‐chain variable fragment (scFv) (derived from Girentuximab) or an EpCAM scFv (clone: MOC31) [17, 37], fused to the hinge and transmembrane domains of human CD8α, along with the intracellular signaling domains of human 4‐1BB and CD3ζ. These sequences were inserted into the lentiviral vector plasmid pRRL, with synthesis carried out by Genscript (Nanjing, China). Lentiviral particles were produced via PEI‐mediated transfection of HEK 293T cells using the CAR‐encoding pRRL plasmid, along with the Δ8.9 packaging construct and the VSVG envelope plasmid. Supernatants containing viral particles were collected 48 h post‐transfection, and viral titers were determined by flow cytometry.

Human peripheral blood mononuclear cells (PBMCs) were isolated using Lymphoprep gradient (07851/07861, StemCell Technologies) from peripheral blood donated by healthy donors and stored in liquid nitrogen for CAR‐T preparation. PBMCs were eventually thawed and activated with Human T‐Activator CD3/CD28 Dyna‐beads at a ratio of 1:1, and 24 h later, PBMCs were infected with the lentivirus encoding various CAR genes generated as described above. The Dyna‐beads were removed on day 5, then T cells were further cultured for other 5 days to proliferation.

In the case of murine‐derived CAR‐T cells, a second‐generation CAR design was also employed. The configuration included an anti‐human CAIX scFv (derived from Girentuximab) or EpCAM scFv (clone: MOC31) linked to the hinge and transmembrane regions of murine CD8α, as well as the cytoplasmic domains of murine 4‐1BB and CD3ζ. An internal ribosome entry site (IRES) was incorporated downstream to enable co‐expression of GFP. The DNA sequence of CAR was inserted into the pMig retroviral vector and the expression is driven by the 5'LTR of the vector. Retroviral particles were generated by transfecting HEK 293T cells with the pMig retroviral vector carrying the CAR constructs and the pCL‐Eco packaging plasmid, using PEI. Viral supernatants were harvested 48 h after transfection, and titers were assessed via flow cytometry.

Murine T cells were isolated from murine lymph node and stimulated with 1 µg/mL anti‐mouse CD3 and 0.2 µg/mL anti‐mouse CD28 monoclonal antibodies (BioLegend, USA) for 24 h. Murine T cells were infected with retrovirus encoding various CAR genes, and further expanded in complete X‐VIVO medium. On day 6‐7, T cells were collected and used for experiments in vitro or in vivo.

### 5.4 Enzyme‐Linked Immunosorbent Assay (ELISA)

After being treated with a kinase inhibitor or not, the CAIX‐CAR‐T cells were treated with recombinant human CAIX for 24 h, the media were collected after centrifugation removal of cell debris for detecting CCL5 levels. After being treated with a kinase inhibitor or not, tumor cells were stimulated by CCL5 for 24 h, the media were collected after centrifugation removal of cell debris for detecting VEGF levels.

Cytokines (CCL5 and VEGF) in supernatants were measured using human ELISA kits. The detection was performed according to the manufacturer's instructions, and the absorbance was detected by Cytation3 (BioTek). Standard curves were illustrated by standards provided by kits. Concentration of cytokines was calculated by multiply dilution ratios.

### 5.5 Flow Cytometry (FACS)

For the analysis of CAR expression of CAR‐T cells, 5 × 10^5^ T cells were collected and washed with PBS twice, Single‐cell samples were incubated with live/dead green dye (Life Technologies, USA) for 30 min at 4°C. Then, cells were stained with murine derived primary antibody against myc‐tag, followed by fluorescent labeled antibodies against murine IgG.

For the analysis of CCL5 expression in tumor‐infiltrating T cells, 1 × 10^6^ cells were collected and washed with PBS twice, Single‐cell samples were incubated with live/dead green dye (Life Technologies) for 30 min at 4°C, followed stained with fluorescent labeled antibodies against murine CD45, CD3, and CD90.1, then cells membrane were permeabilized and stained using a flow cytometry antibody specific for CCL5. Cells were fixed with 1% paraformaldehyde (VIH130, VICMED). Samples were analyzed by using fluorescence‐activated cell sorter (BD FACS Canto II) and analyzed by FlowJo software (Tree Star, San Carlos, CA).

### 5.6 Real‑Time Cell Analysis (RTCA)

The cytotoxicity of CAR‐T cells in vitro was determined using the Agilent xCELLigence RTCA instrument according to a protocol described earlier [38, 39]. In brief, the background impedance was measured by adding 50 µL of the cell culture medium to an E‐plate (ACEA Biosciences). Tumor cells (3000–5000 cells/well) were seeded and allowed to adhere. Flow‑sorted tumor‑infiltrating CAR‑T cells were then added at an effector‑to‑target (E:T) ratio of 10:1. Target‑only wells served as proliferation controls. The CAR‐T cytotoxic activity was determined based on attached viable target cells (cell index value). Data are shown after normalizing the cell index with the time point of CAR‐T cell addition (normalized cell index).

### 5.7 Immunofluorescence (IF)

For Ki67 staining, PFA‑fixed tumor tissues were paraffin‑embedded and sectioned at 5 µm. After baking at 60°C, slides were deparaffinized and rehydrated through graded ethanol (99 %, 95 %, 70 %). Antigen retrieval was performed by microwaving in sodium citrate buffer (Proteintech, PR30001) for 20 min. Sections were blocked with 3 % BSA in PBS, incubated with a rabbit anti‑Ki67 primary antibody (abcam, ab16667), and then stained with a CoraLite Plus 594‑conjugated goat anti‑rabbit recombinant secondary antibody (1:400, Proteintech, RGAR004). Nuclei were counterstained with DAPI using mounting medium (VICMED, VS1008). Images were acquired on a fluorescence microscope (ZEISS Axio Observer 7, LSM880), and Ki67‑positive cells were quantified with ImageJ to determine the percentage of Ki67‑positive cells.

For TUNEL staining, tumor tissue sections were baked at 60°C, deparaffinized, and rehydrated through a graded ethanol series (99 %, 95 %, 70 %). Slides were then incubated with 40 µg/mL proteinase K at room temperature for 20 min, followed by labeling with TUNEL reaction mixture (Proteintech, PF00006) at 37°C for 2 h. After washing, sections were mounted with DAPI‑containing mounting medium and imaged under a fluorescence microscope. TUNEL‑positive cells were quantified using ImageJ.

### 5.8 Protein Extraction and Western Blotting

Tumor tissues were washed with ice‐cold PBS and disassociated using an ultrasonic cell disruptor, or cells were washed thrice with ice‐cold PBS, and lysed at 4°C for 30 min with a lysis buffer (50 mM Tris·HCl, pH 7.5, 150 mM NaCl, 1% Triton X‐100, 0.5% deoxycholate, 0.1% SDS, 10% glycerol, 1 mM EDTA, 1 mM EGTA) containing a protease inhibitor cocktail (Thermo Scientific, USA), the cell lysates were clarified by centrifugation (12,000 g, 10 min, 4°C), and supernatant was collected and stored at ‐40°C or used for immunoblotting.

Protein samples were separated by SDS‐PAGE after quantification with BCA kits (KGB2101, KeyGEN BioTECH) and transferred to nitrocellulose membranes (GE Healthcare, USA). The membranes were blocked with 5% non‐fat milk in Tris‐buffered saline with Tween‐20 (TBS‐T) for 1 h at room temperature and then incubated with primary antibodies as indicated in 2% BSA in TBS‐T overnight at 4°C followed by incubation with horseradish peroxidase‐conjugated secondary antibodies and analyzed by chemiluminescence with ECL.

### 5.9 RNA Isolation and Real‐Time Quantitative PCR (Q‐PCR)

Total RNA from tumor tissues or cells was extracted using the TRIzol reagent (VR603, VICMED), and the concentration was measured using NanoDrop 2000. RNA (500 ng) was used for reverse transcription using the PrimeScript RT Master Mix (RR036, Takara). Meanwhile, gene expression was detected via qRT‐PCR using the SYBR Green reagent (Q111‐02, Vazyme) on LightCycler 96 (Roche), with β‐actin as the control for normalization. The relative gene expression was analyzed using 2^−△△CT^. The information of primer sequences is provided in Table S3.

### 5.10 Bulk mRNA Sequencing and Gene Set Enrichment Analysis (GSEA)

Total RNA from sorted cancer cells for tumor tissues, or CAR‐T cells was extracted using the TRIzol reagent following the manufacturer's instructions. Next, RNA was sequenced as described previously [39]. In brief, mRNA was purified from total RNA (5 µg) using Dynabeads Oligo (dT) (Thermo Fisher, CA, USA) and fragmented into short fragments using divalent cations with the Magnesium RNA Fragmentation Module (NEB, MA, USA). The cleaved RNA fragments were reverse‐transcribed to form cDNA with SuperScript II Reverse Transcriptase (Invitrogen, CA, USA). The cDNA was used to synthesize U‐labeled double‐stranded DNA with *Escherichia coli* DNA polymerase I (NEB, MA, USA), RNase H (NEB, MA, USA), and dUTP solution (Thermo Fisher, CA, USA). Finally, 2 × 150 bp paired‐end sequencing (PE150) was performed on the Illumina NovaSeq 6000 (LC‐Bio Technology Co., Ltd., Hangzhou, China) following the vendor's recommended protocol.

GSEA was performed using the GSEA (v4.1.0) and MSigDB software to identify a set of genes in specific KEGG pathways that were significantly differently expressed in various treatments. The enrichment scores and *p* value were calculated with default parameters. KEGG pathways with NES > 1 or NES < −1 and NOM *p*‐value < 0.05 were considered significant in the two groups.

### 5.11 Tissue Dissociation and Preparation of Single‐cell Suspensions

Single‐cell suspensions were prepared from tumor tissues as previously described [40]. Briefly, tissues were dissected in a sterile RNase‐free dish containing ice‐cold calcium‐ and magnesium‐free PBS, minced into ∼0.5 mm^3^ pieces, and washed to remove blood and fat. Tissue dissociation was performed using an enzymatic solution, yielding suspensions with >85% viability (trypan blue exclusion). Cell concentration was adjusted to 700–1200 cells/µL (Countess II Automated Cell Counter) for downstream analysis.

### 5.12 10× Genomics Chromium Library and Sequencing

Single‐cell suspensions were loaded to 10× Chromium to capture 5000 single cells according to the manufacturer's instructions of 10× Genomics Chromium Single‐Cell 3’ kit (V3). cDNA amplification and library construction steps were performed according to the standard protocol. Libraries were sequenced on an Illumina NovaSeq 6000 sequencing system (paired‐end multiplexing run,150 bp) by LC‐Bio Technology co.ltd., (HangZhou,China) at a minimum depth of 20,000 reads per cell.

### 5.13 Tumor Models and Treatment

Male NOD‐*Prkdc^scid^Il2rg^null^* (NPG) mice (aged 6–8 weeks) were purchased from Vitalstar Biotechnology (Beijing, China). Male C57BL/6N mice (aged 6–8 weeks) were purchased from Animal Center of Xuzhou Medical University (Xuzhou, China). C57BL/6N‐CCL5^flox/+^ mice were purchased from Cyagen (Soochow, China). Cd4‐CreERT2 mice were purchased from were purchased from Shanghai Model Organisms Center (Shanghai, China). Mice were maintained in specific pathogen‐free animal facilities of the Experimental Animal Center of Xuzhou Medical University (Xuzhou, China). All animal experiments were approved by the Animal Ethics Committee of Xuzhou Medical University (ethics approval number: 202301T002).

For subcutaneous tumor models in immunocompromised mice, 2 × 10^5^ B16‐F10^hCAIX+^, or 1 × 10^6^ MC38^hEpCAM+^ cancer cells were injected into the right dorsal flank region of C57BL/6N mice. These mice were randomly assigned to different groups on day 9, and treated with 100 mg/kg Cyclophosphamide (Cy). 24 h later, mice were injected with 1 × 10^6^, 2 × 10^6^, 5 × 10^6^ of respective murine CAR‐T cells via a tail vein. Tumor growth was recorded every 2–3 days, the single‐cell suspension of tumor cells were isolated at the end point of experiments as previously described [17]. Briefly, tumors were cut into pieces and added into a digestion solution: 1 mg/mL Collagenase I (VIC079, VICMED) and 100 U/mL DNase I (10104159001, Roche). Tumor samples were transferred into gentleMACS C tubes and processed into single‐cell suspensions using a Miltenyi gentleMACS dissociator with heaters (37°C). CCL5 expression of the tumor cells and tumor infiltrated T cells were analyzed by FACS.

For subcutaneous tumor models in immunodeficient mice, 2 × 10^6^ OSRC‐2, or 1 × 10^6^ HCT116 cancer cells were injected into the right dorsal flank region of NSG mice. These mice randomly assigned to different groups on day 7, were injected with 2 × 10^6^, 6 × 10^6^, and 2 × 10^7^ of respective human‐derived CAR‐T cells via a tail vein. Tumor growth was recorded every 2–3 days. Protein and mRNA samples from tumor tissues were extracted as previous indicated.

In all mouse tumor models, tumor size did not exceed 10% of the animal's body weight, and tumor volume did not exceed 2000 mm^3^ (20 mm in diameter) at any point during the study. Additionally, animal body weight loss did not exceed 15% of the original body weight at any time during the study, and the mouse body weight monitoring data were provided in the Supporting Information.

### 5.14 Whole‐Mount Staining for Tumor Vasculatures Detection

Part of tumor tissues were collected at the end point of subcutaneous tumor models were subjected to whole mount staining for tumor vasculature visualization as previous report [41]. Briefly, tumor tissues were fixed overnight with 4% Paraformaldehyde (VIH130, VICMED), then cut into thin slices and incubated for 5 min with 20 mM proteinase K in PBS buffer. After incubation with methanol for 30 min, tumor slice samples were washed twice in PBS, followed by overnight incubation with 3% non‐fat milk in 0.3% Triton X‐100 in PBS. Tumor samples were incubated overnight with a goat anti CD31 antibody (1:200; AF3628, R&D Systems), followed by the 2 h incubation of Rhodamine (TRITC)–conjugated Donkey Anti‐Goat IgG(H+L) (1:400, SA00007‐3, Proteintech). After washing in PBS, samples were mounted using a Antifluorescence quenching mounting medium (VS1008, VICMED) and positive signals were captured using confocal microscopy (LSM880, Zeiss, Germany) equipped with ZEISS ZEN 3.6 software.

### 5.15 Clinical Trials

The clinical trial was sponsored and designed by the Affiliated Hospital of Xuzhou Medical University, and was approved by the Medical Ethics Committee of Xuzhou Medical University Affiliated Hospital (Approved number: XYFY2021‐KL325‐01 for Digestive system tumors, XYFY2021‐KL272‐02 for Ovarian cancer, and XYFY2022‐KL170‐01 for Urinary system tumors, XYFY2021‐KL317‐02 for Nasopharyngeal carcinoma). Enrollment occurred from February 2022 to September 2023. Five patients aged 50–70 years were enrolled, including one with gastric cancer, one with ovarian cancer, and two with prostate cancer. Above four patients underwent leukapheresis for the preparation of B7H3‐targeted CAR‐T cells. One patient with metastatic nasopharyngeal carcinoma was enrolled and underwent leukapheresis for CAR‐T preparation targeting gp350 antigen. The characteristics of the patients were provided in Table S4. All patients enrolled and treated in this trial signed written informed consents prior to participation. All clinical investigations were conducted in accordance with the Declaration of Helsinki principles.

### 5.16 Statistical Analysis

Data are represented as the mean ± S.D. All data have been generated form at least three independent biological experiments, unless otherwise specified in the figure legends. Prior to analysis, normality and homogeneity of variance tests were performed. Two‐tailed unpaired student's *t*‐test were used for comparisons between two unpaired group, while one‐way (for a single factor) ANOVA was employed for multi‐group comparisons. Tumor growth curves were analyzed using two‐way repeated‐measures ANOVA. In call cases, a significant result was defined as *p* < 0.05. Statistical analysis was performed using GraphPad Prism 8.1 software (CA, USA). The sample size (*n*) and statistical methods for each experiment are indicated in the corresponding figure legends. All data are available in the article and Supporting Information.

## Author Contributions

Conceptualization: Q.Z.; Methodology: S.S., G.W., X.L.; Software: X.G., H.X.; Validation: S.S., Q.L., B.W.; Formal analysis: J.S., H.L., L.L.; Investigation: S.S., Q.L., B.W., G.W., Y.X., Y.L., C.H., Z.W.; Resources: Q.Z., S.S.; Data curation: S.S., Q.L.; Writing – original draft preparation: S.S.; Writing – review and editing: Q.Z.; Visualization: X.L., P.M.T., Q.Z.; Supervision: Q.Z.; Project administration: Q.Z.; Funding acquisition: Q.Z., S.S., J.S., H.L.

## Conflicts of Interest

The authors declare no conflicts of interest.

## Supporting information




**Supporting File**: advs75551‐sup‐0001‐SuppMat.docx.

## Data Availability

The data that support the findings of this study are available from the corresponding author upon reasonable request.
