# Supplementary material for: Enhancing CAR‐T Cell Efficacy in Solid Tumors by Inhibiting CCL5/VEGF‐Mediated Angiogenesis
Source: Adv Sci (Weinh). 2026 May 6;13(42):e21975. doi: 10.1002/advs.202521975 (PMC13336079; doi:10.1002/advs.202521975)
Supplement: Supplementary file 1 — Supporting File: advs75551‐sup‐0001‐SuppMat.docx. [file ADVS-13-e21975-s001.docx]

**Supplementary Figures and Legends**


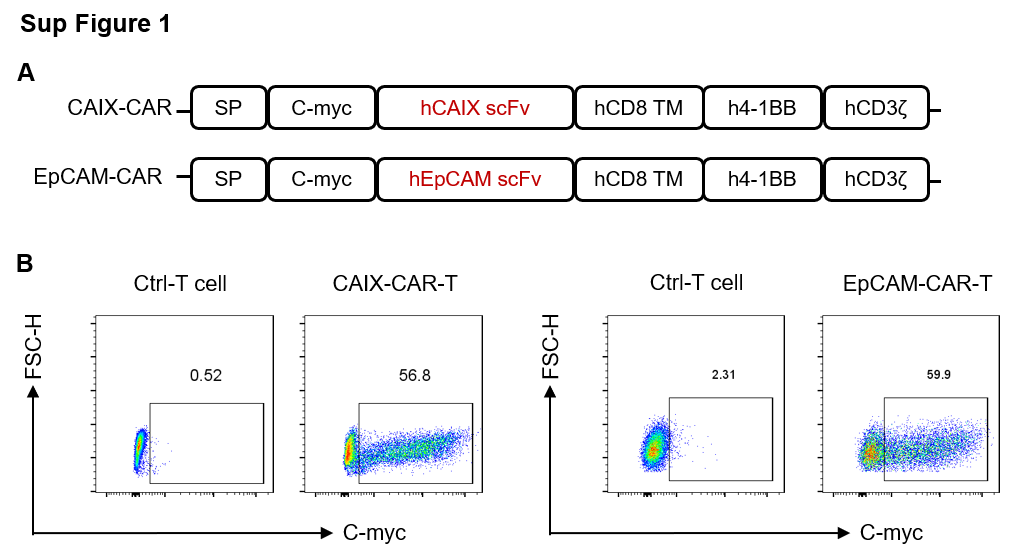


**Supplementary Figure S1. Preparations of human derived CAR T cells targeting human CAIX or EpCAM antigen. A.** Schematic of second-generation CAR constructs comprising anti-human CAIX or EpCAM scFv, human 4-1BB costimulatory domain (CSD), and CD3ζ signaling domain. **B.** Flow cytometry analysis of CAR expression.


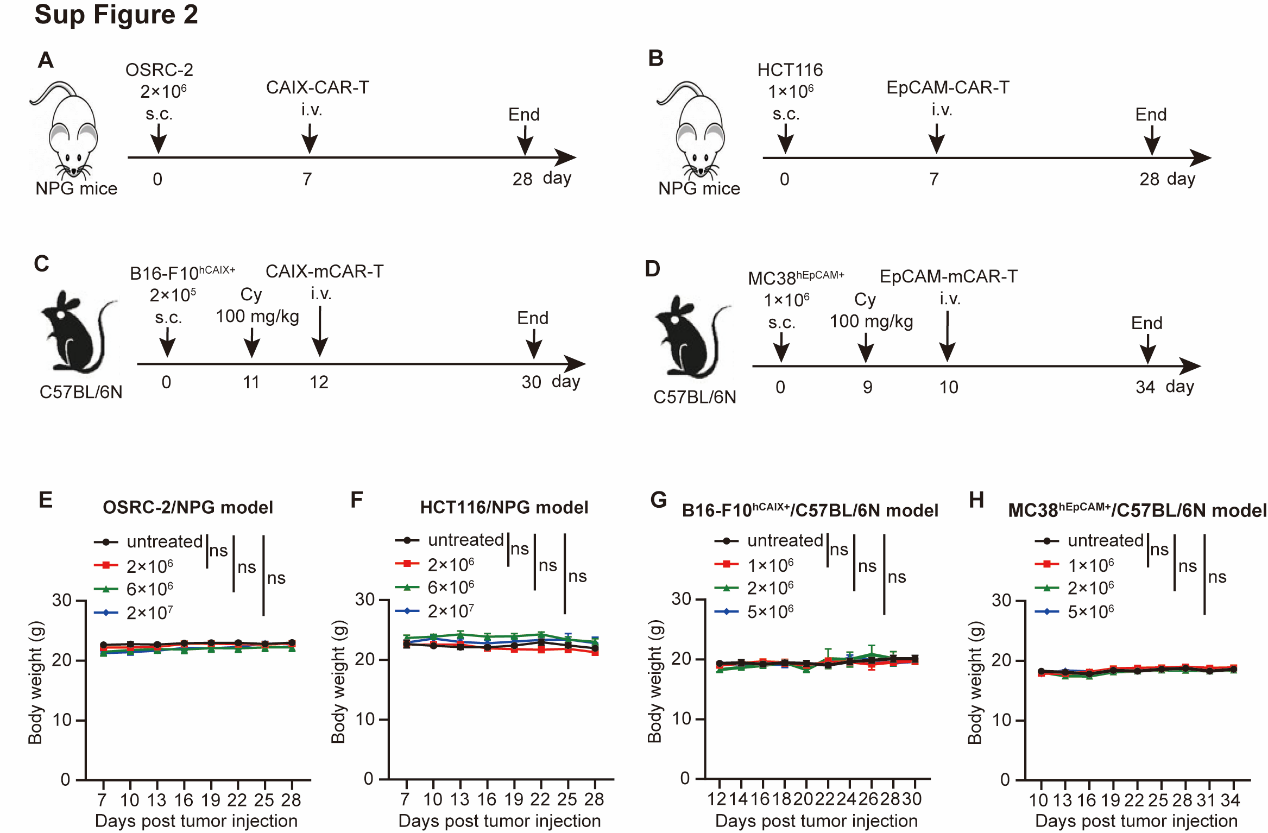


**Supplementary Figure S2. Schematic diagram of the experimental design and mouse body weight monitoring (related to Figure 1). A.** NPG mice (NOD-*Prkdc^scid^Il2rg^null^*) were subcutaneously injected with OSRC-2 cells (2×10⁶) to establish the xenograft model; Mice were randomly assigned to one of four groups (n = 6 each) and treated with 2×10⁶, 6×10⁶, or 2×10⁷ CAIX-CAR-T cells on day 7; Experiments were terminated on day 28. **B.** NPG mice were subcutaneously injected with HCT116 cells (1×10⁶) to establish the xenograft model; Mice were randomly assigned to one of four groups (n = 6 each) and treated with 2×10⁶, 6×10⁶, or 2×10⁷ EpCAM-CAR-T cells on day 7; Experiments were terminated on day 28. **C.** C57BL/6N mice were subcutaneously injected with B16-F10^hCAIX+^ cells (2×10^5^), preconditioned with Cyclophosphamide (Cy) on day 11, and treated on day 12 with CAIX-mCAR-T cells (1×10⁶, 2×10⁶, or 5×10⁶) or left untreated (n=6/group); Experiments were terminated on day 30. **D.** C57BL/6N mice were injected with MC38^hEpCAM+^ cells (1×10⁶), preconditioned with Cy on day 9, and treated on day 10 with EpCAM-CAR-T cells (1×10⁶, 2×10⁶, or 5×10⁶) or left untreated (n=6/group); Experiments were terminated on day 34. **E-H.** Mouse body weights of various tumor models recorded during the experiment. (E) OSRC-2 tumor model, (F) HCT116 tumor model, (G) B16-F10^hCAIX+^ tumor model, (H) MC38hEpCAM+ tumor model. Data were analyzed by two-way repeated-measures ANOVA followed by Dunnett’s multiple-comparisons test. ns, no significance for indicated comparison.


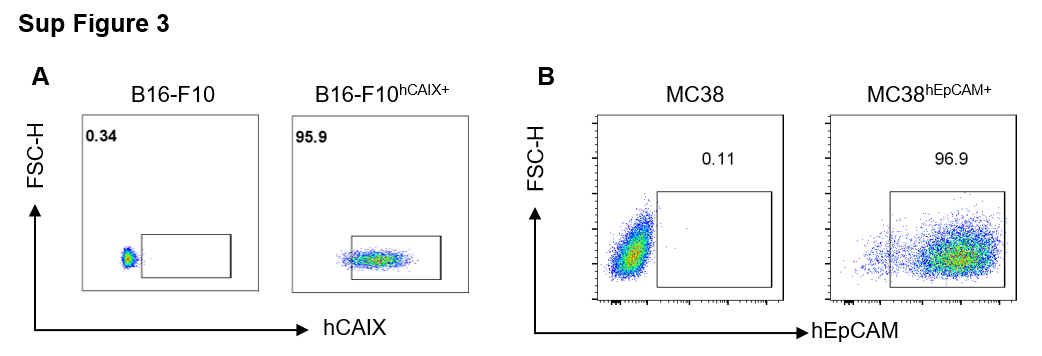


**Supplementary Figure S3.** Generation of B16-F10 and MC38 cells overexpressing human CAIX or EpCAM. Stable overexpression of human CAIX in B16-F10 or EpCAM in MC38 was achieved as described in *Methods*. **A.** Flow cytometry analysis of human CAIX expression in B16-F10. **B.** Flow cytometry analysis of human EpCAM expression in MC38.


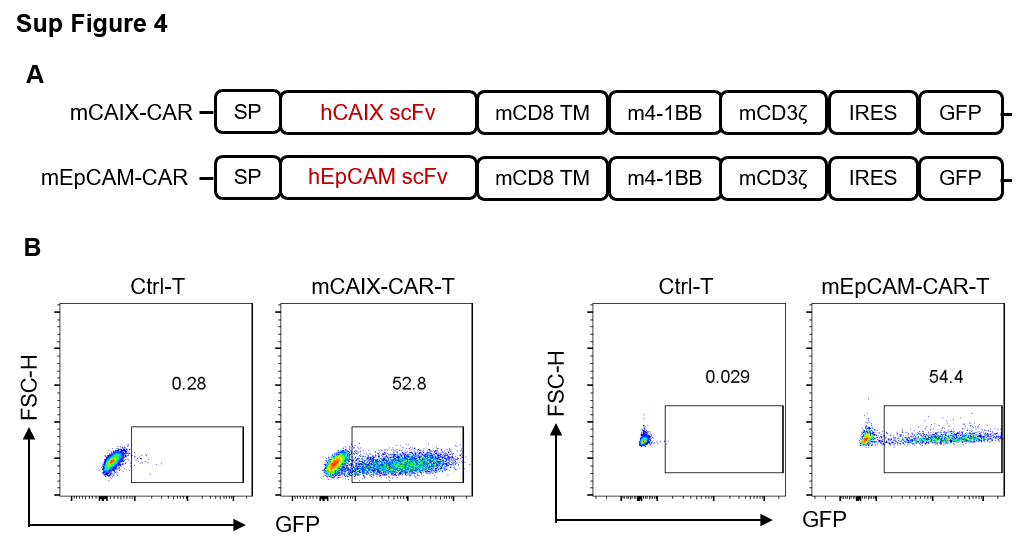


**Supplementary Figure S4. Preparations of murine derived CAR T cells targeting human CAIX or EpCAM antigen. A.** Schematic of second-generation CAR constructs containing anti-human CAIX or EpCAM scFv, murine 4-1BB costimulatory domain (CSD), and CD3ζ signaling domain. GFP was included for CAR expression tracking. **B.** Flow cytometry analysis of CAR expression (GFP+).


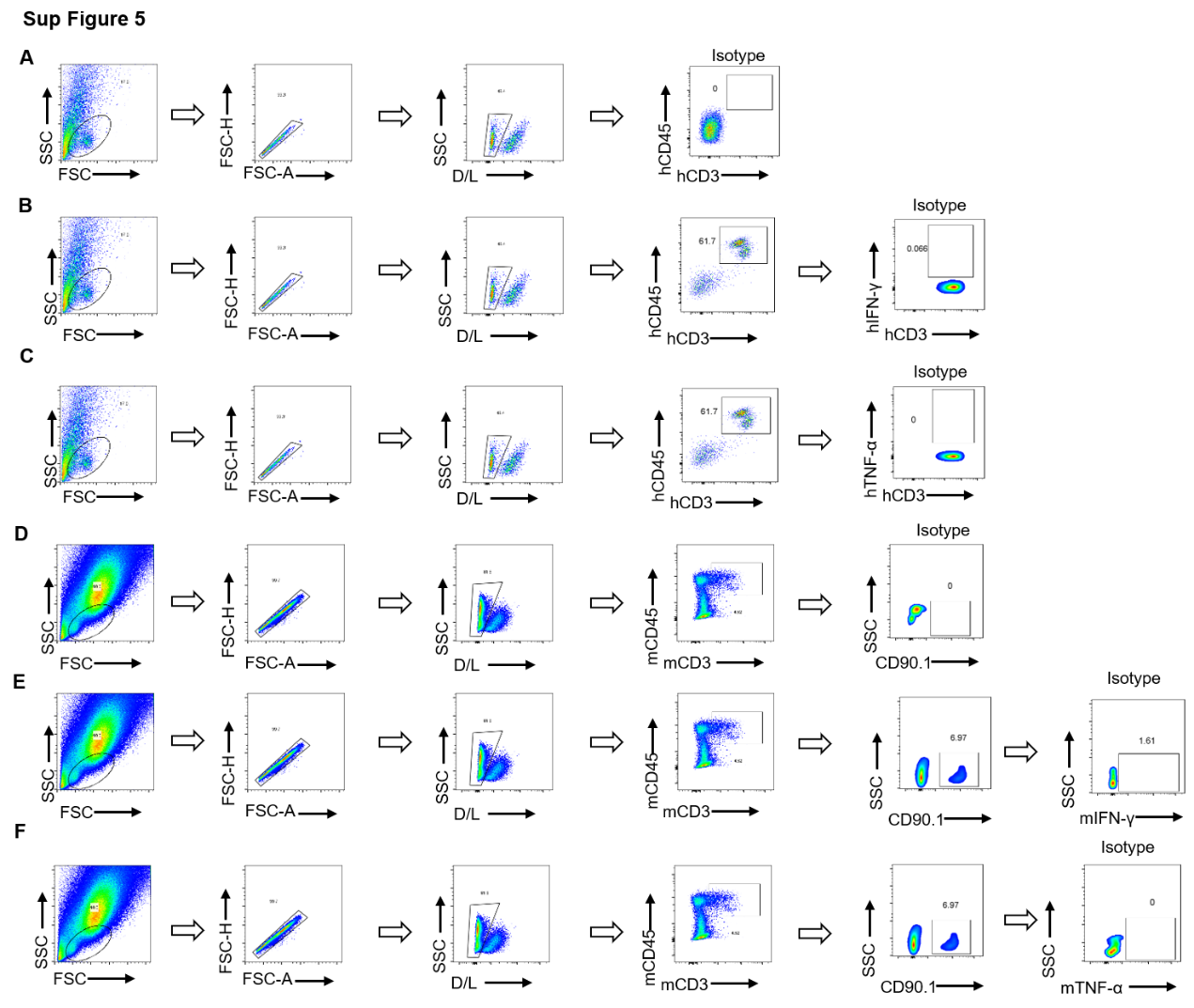


**Supplementary Figure S5. Gating strategies for adoptive T cells and cytokine analysis (related to Figure 1). A-C**. OSRC-2 tumor model: (A) adoptive T cells, IFN-γ (B), and TNF-α (C) analysis. **D-F**. MC38^hEpCAM+^ tumor model: (D) adoptive T cells, IFN-γ (E), and TNF-α (F) analysis.


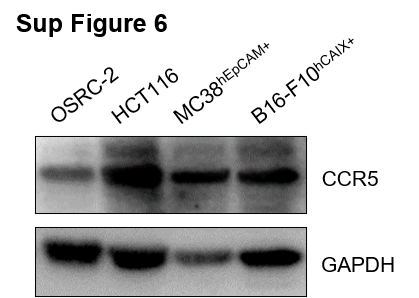


**Supplementary Figure S6. Western blot analysis of CCR5 expression in tumor cell lines.** Protein lysates from OSRC‑2, HCT116, MC38^hEpCAM+^, and B16^hCAIX+^ cells were subjected to immunoblotting to determine CCR5 protein levels.


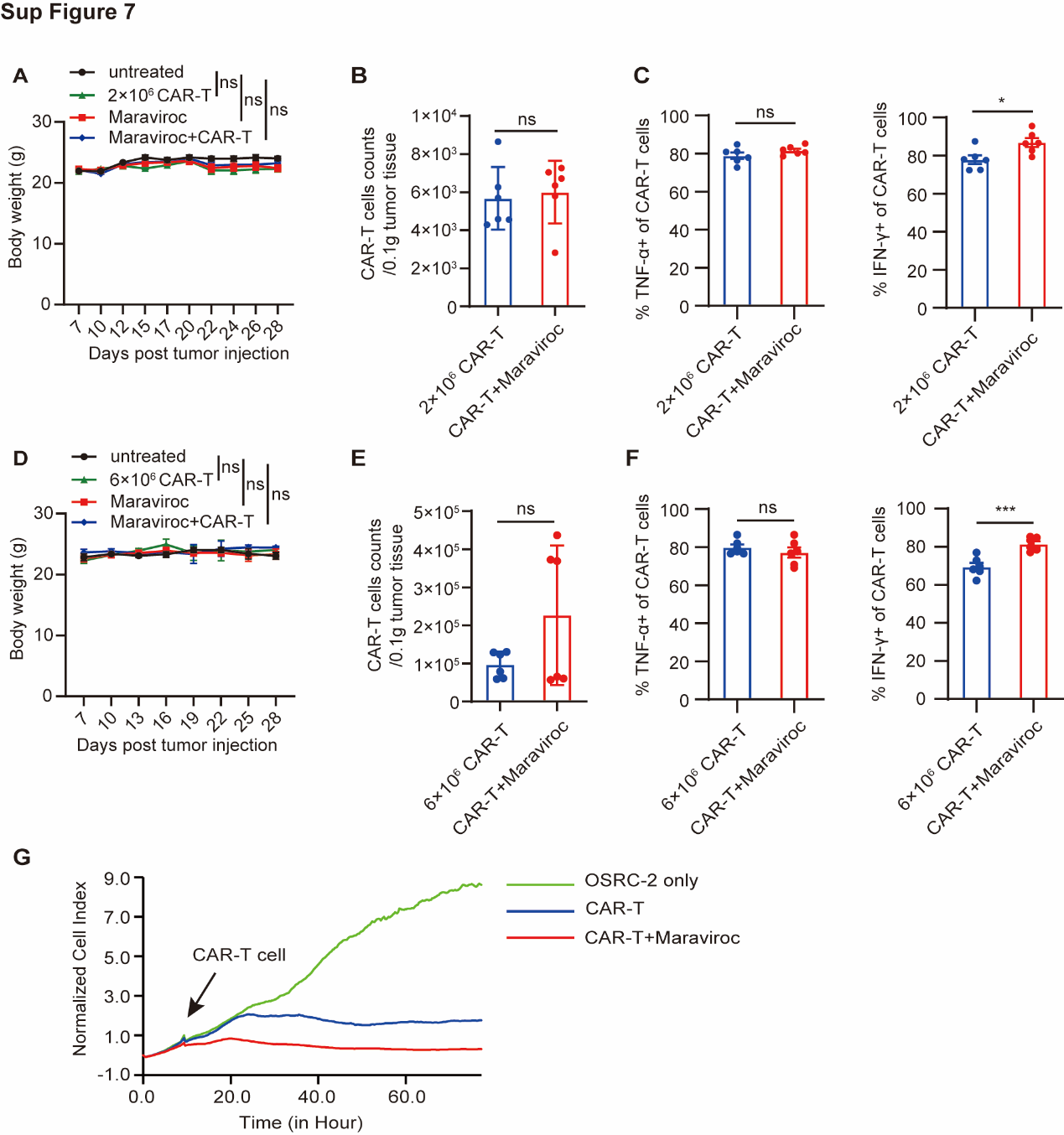


**Supplementary Figure S7. Maraviroc does not impair the tumor infiltration or effector function of CAR‑T cells in an immunodeficient OSRC‑2 xenograft model (related to Figure 2).** **A-C.** NPG mice bearing OSRC‑2 xenografts (2×10⁶ cells) were treated with Maraviroc (15 mg/kg) from day 6, followed by administration of 2×10⁶ CAIX‑specific CAR‑T cells (n=6 per group). Mouse body weights were recorded during the experiment (A). Tumors in CAR-T and CAR-T combine Maraviroc treatment group were harvested three weeks later and dissociated into single‑cell suspensions. After 6‑hour stimulation with PMA, Ionomycin, and BFA, tumor‑infiltrating CAR‑T cells (hCD45⁺hCD3⁺) were analyzed by flow cytometry for the quantity of tumor-infiltrating CAR‑T cells (B) as well as the expression of cytokines TNF‑α and IFN‑γ (C). **D-G.** NPG mice bearing OSRC‑2 xenografts (2×10⁶ cells) were treated with Maraviroc (15 mg/kg) from day 6, followed by administration of 6×10⁶ CAIX‑specific CAR‑T cells (n=6 per group). Mouse body weights were recorded during the experiment (D). Tumors in CAR-T and CAR-T combine Maraviroc treatment group were harvested three weeks later and dissociated into single‑cell suspensions. After 6‑hour stimulation with PMA, Ionomycin, and BFA, tumor‑infiltrating CAR‑T cells (hCD45⁺hCD3⁺) were analyzed by flow cytometry for the quantity of tumor-infiltrating CAR‑T cells (E) as well as the expression of cytokines TNF‑α and IFN‑γ (F). Flow‑sorted tumor‑infiltrating CAR‑T cells were co‑cultured with tumor cells at a 10:1 ratio in a RTCA assay to evaluate cytotoxic activity (G). Above tumor tissues were collected from animal experiments in Figure 2G-M. Data (A, D) were analyzed by two-way repeated-measures ANOVA followed by Dunnett’s multiple-comparisons test. Other data were represented as mean ± SD and were analyzed using t-test. *, P < 0.05; ***, P < 0.001; ns, no significance for indicated comparison.


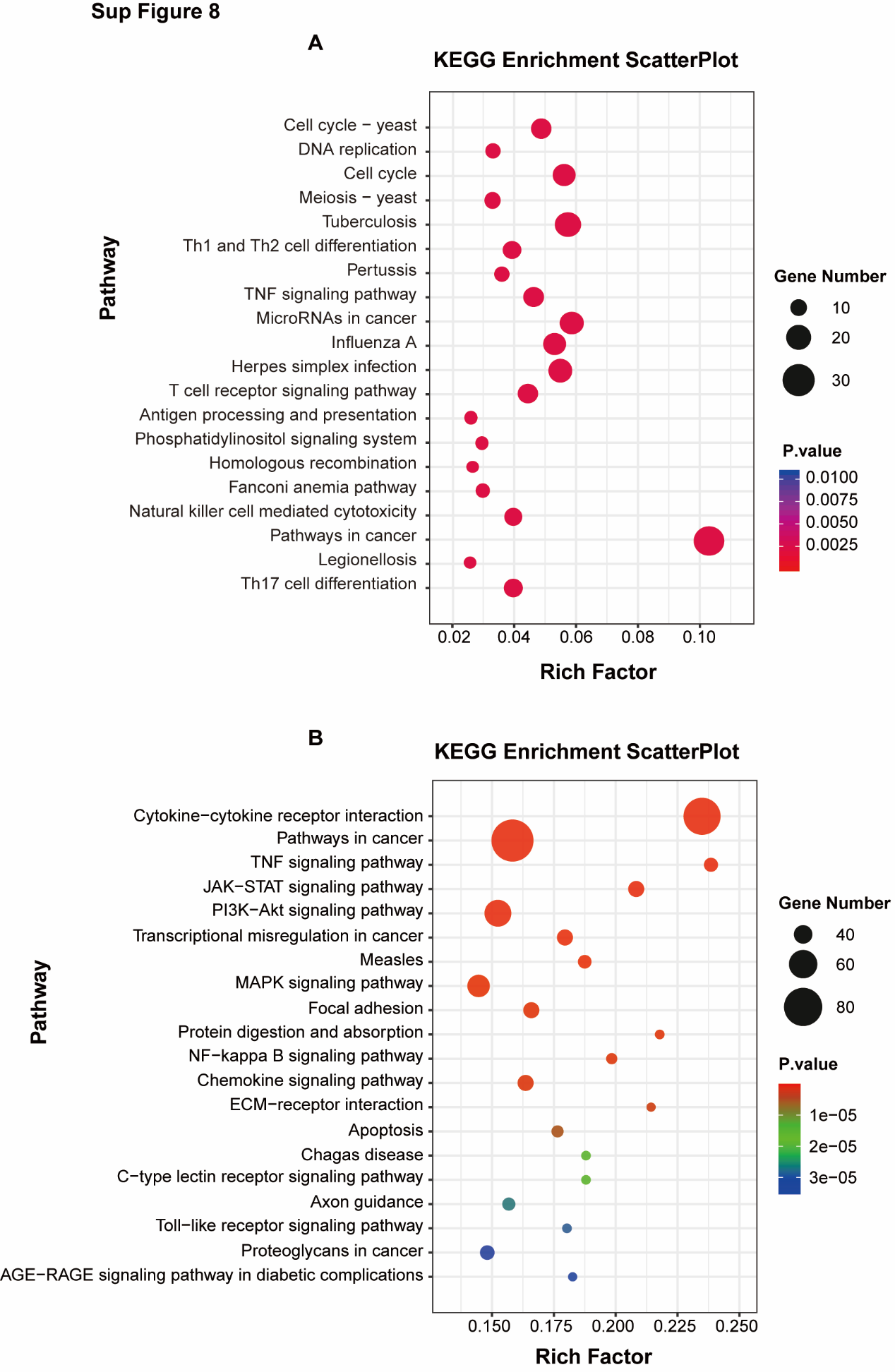


**Supplementary Figure S8. KEGG enrichment analysis of enriched pathways. A**. KEGG enrichment analysis of pathways upregulated in tumor cells following CAR-T cell therapy. Tumor cells were sorted from untreated mice or mice treated with 2×10⁶ CAR-T cells (n = 6 per group), pooled, and subjected to RNA sequencing with two technical replicates. The tumor treatment procedure is shown in Supplementary Fig. S2. Top 20 pathways are shown. **B**. KEGG enrichment analysis of pathways enriched in CAIX-CAR-T cells upon tumor antigen stimulation. CAIX-CAR-T cells were stimulated with the tumor antigen rhCAIX for 24 hours in vitro. Stimulation was performed in two separate batches, followed by RNA sequencing. Top 20 pathways are shown.


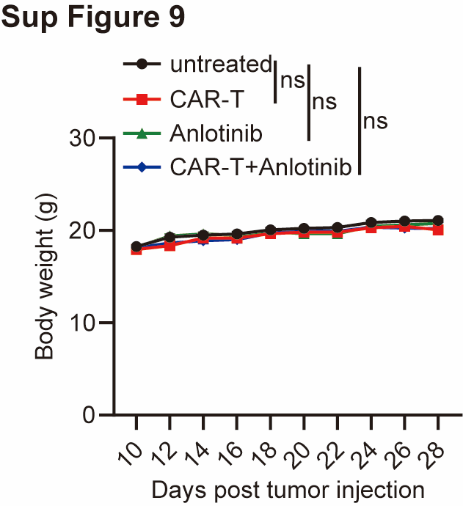


**Supplementary Figure S9. Mouse body weight recording results (related to Figure 3).** C57BL/6 mice bearing MC38^hEpCAM+^ xenografts (1×10⁶ cells) received anlotinib (0.5 mg/kg) from day 10, followed by administration of EpCAM-CAR-T cells (1×10⁶; n=5/group). i.g., intragastric administration. Mouse body weights were recorded during the treatment process. Data were analyzed by two-way repeated-measures ANOVA followed by Dunnett’s multiple-comparisons test. ns, no significance for indicated comparison.


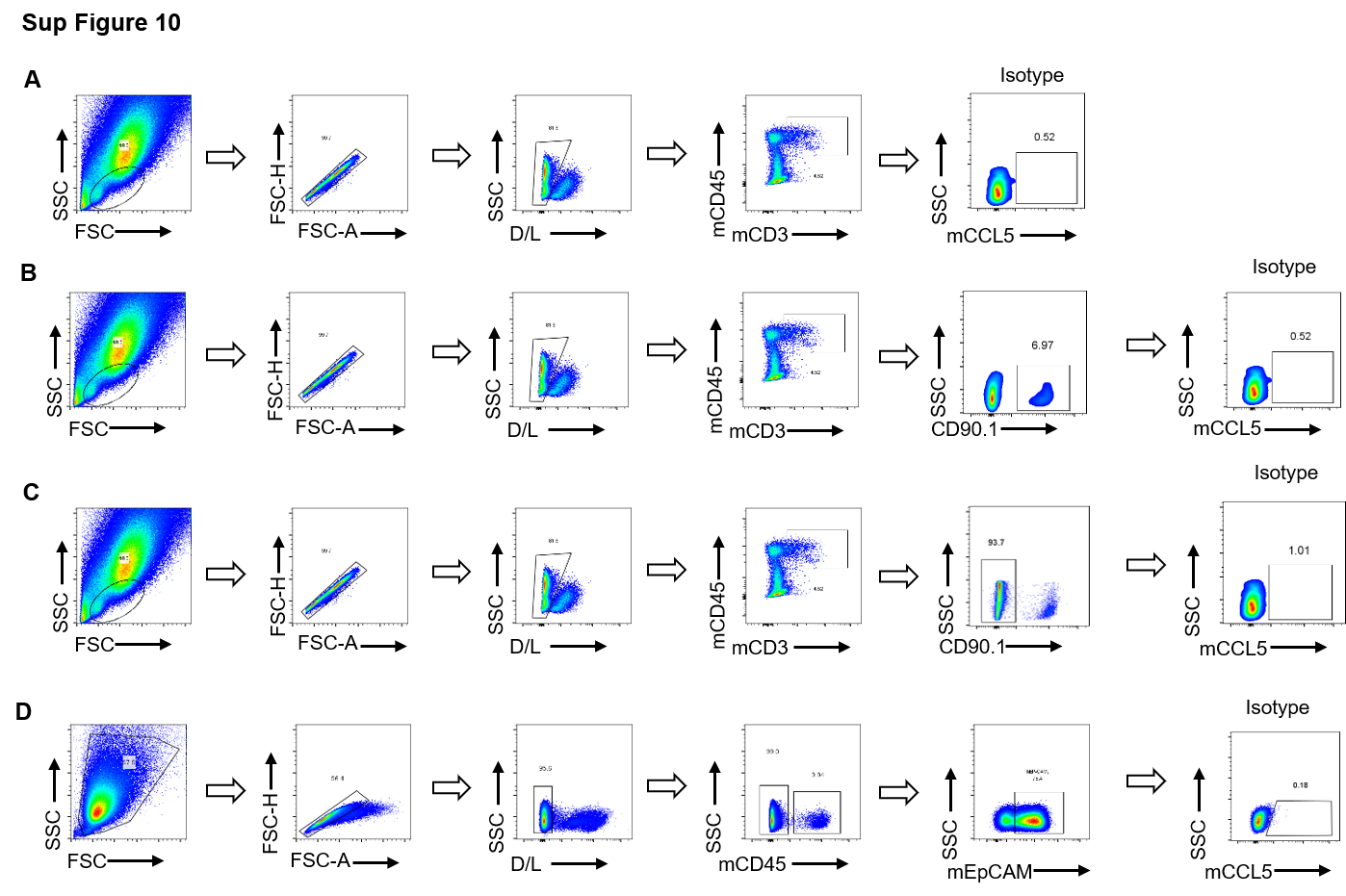


**Supplementary Figure S10. Gating strategies for CCL5⁺ analysis in various cell types in tumor tissues (related to Figure 4). A**. Gating strategy for tumor-infiltrating CD3⁺ T cells. **B**. Gating strategy for CD3⁺, CD90.1⁺ CAR-T cells. **C**. Gating strategy for CD3⁺, CD90.1⁻ endogenous T cells. **D**. Gating strategy for tumor cells.


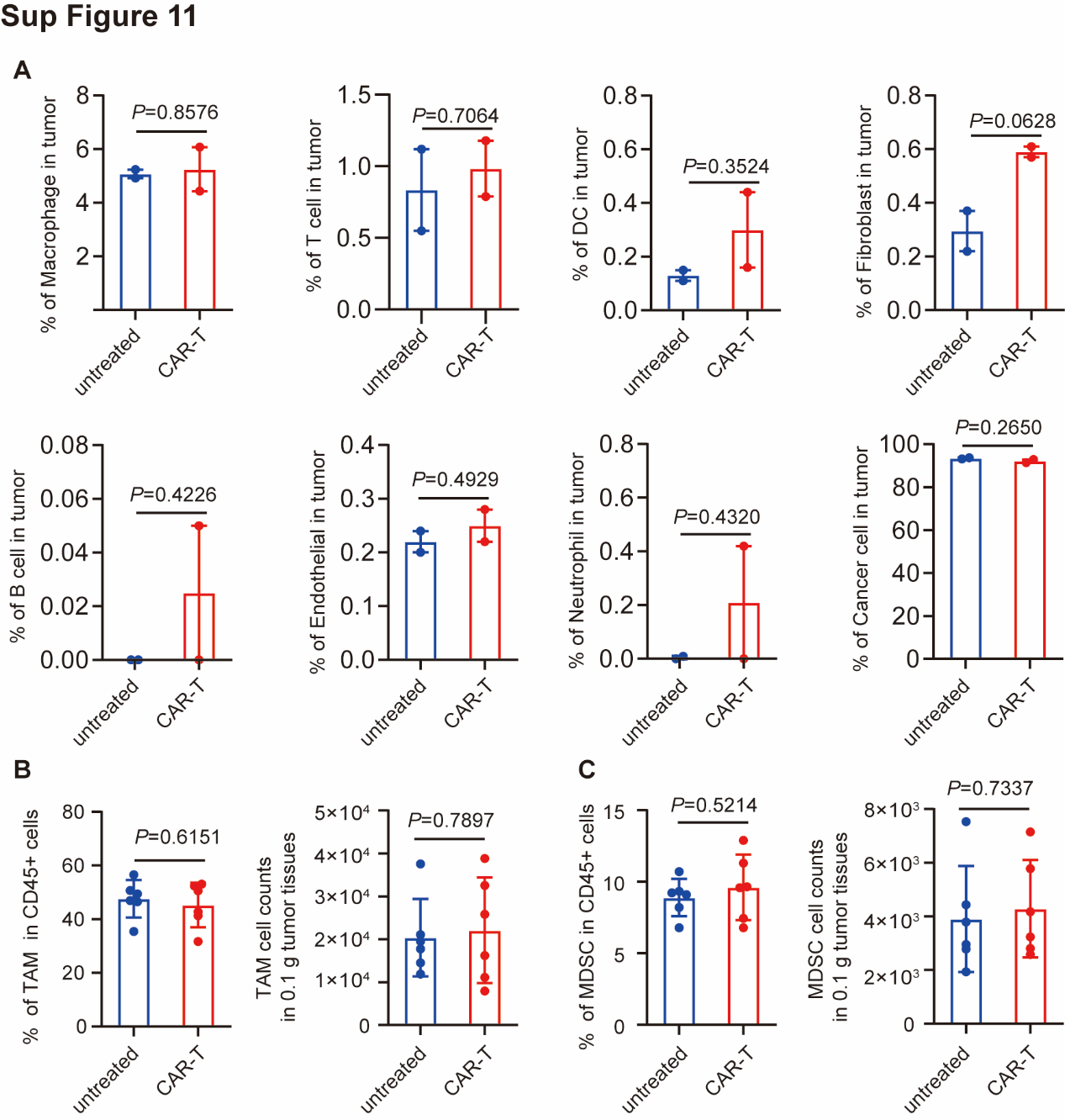


**Supplementary Figure S11. Low‑dose CAR‑T therapy does not significantly alter the tumor immune microenvironment. A.** Single‑cell RNA sequencing analysis of MC38^hEpCAM+^ tumors from C57BL/6N mice treated with EpCAM‑specific CAR‑T cells. Tumor‑bearing mice (1×10⁶ MC38^hEpCAM+^ cells) received cyclophosphamide (day 9) followed by CAR‑T cell infusion (day 10). scRNA‑seq was performed at week 4, and the proportions of macrophages, T cells, dendritic cells, B cells, neutrophils, fibroblasts, endothelial cells, and tumor cells within the tumor tissue were quantified. **B, C.** Flow cytometry analysis comparing the proportion and absolute number of tumor‑associated macrophages (TAMs) (B) and myeloid‑derived suppressor cells (MDSCs) (C) in untreated versus low‑dose (1×10⁶) CAR‑T‑treated group. Two tumor tissues were collected from each group for scRNA-seq. Data were represented as mean ± SD and were analyzed using the t-test.


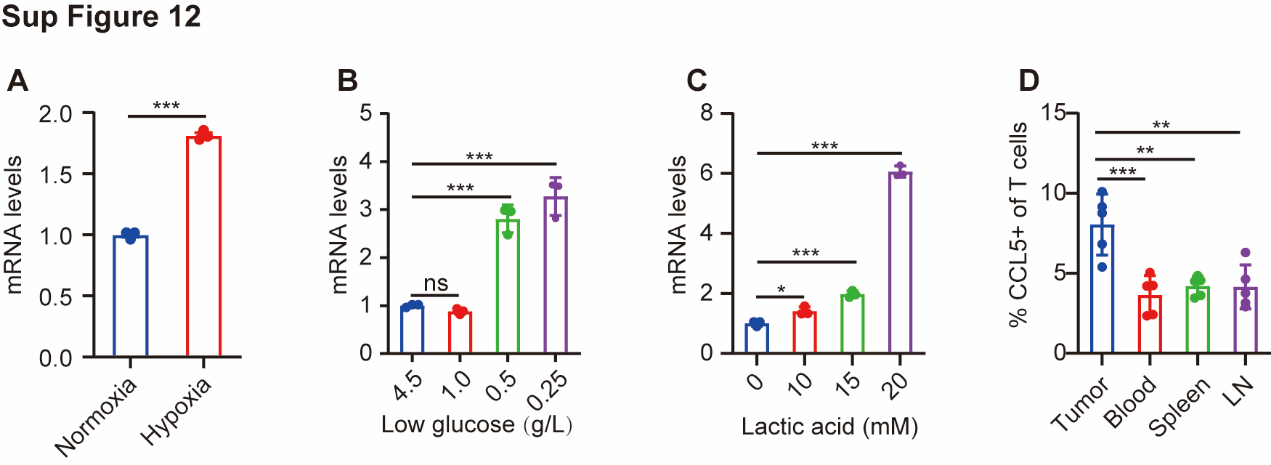


**Supplementary Figure S12. Hypoxic, low‑glucose, and lactic acid conditions in the tumor microenvironment promote CCL5 expression in T cells. A.** CAR‑T cells cultured under hypoxia for 24 hours were analyzed by qPCR for Ccl5 expression. **B.** CAR‑T cells cultured in media containing different glucose concentrations for 24 hours were analyzed by qPCR for Ccl5 expression. **C.** CAR‑T cells cultured in media supplemented with varying concentrations of lactic acid for 24 hours were analyzed by qPCR for Ccl5 expression. **D.** CCL5 expression levels in T cells from the peripheral blood, spleen, draining lymph nodes (LN), and tumor tissue of tumor‑bearing mice were assessed by flow cytometry (n=5). Data (A) were represented as mean ± SD and were analyzed using the t-test. Other data were represented as mean ± SD and were analyzed using one-way ANOVA. *, P < 0.05; **, P < 0.01; ***, P < 0.001; ns, no significance for indicated comparison.


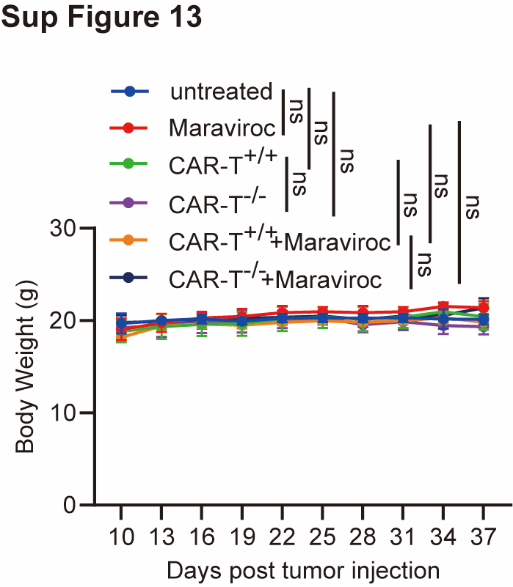


**Supplementary Figure S13. Mouse body weight recording results (related to Figure 6).** Thy-1.1 mice were inoculated with 1×10⁶ tumor cells, treated with cyclophosphamide (day 9), and randomized to six groups (n=6/group): untreated control, Maraviroc (15 mg/kg), wild-type CAR-T (CAR-T^+/+^), Ccl5^-/-^ CAR-T (CAR-T^-/-^), CAR-T^+/+^ + maraviroc, or CAR-T^-/-^ + maraviroc. Mouse body weights were recorded during the treatment process. Data were analyzed by two-way repeated-measures ANOVA followed by Dunnett’s multiple-comparisons test. ns, no significance for indicated comparison.


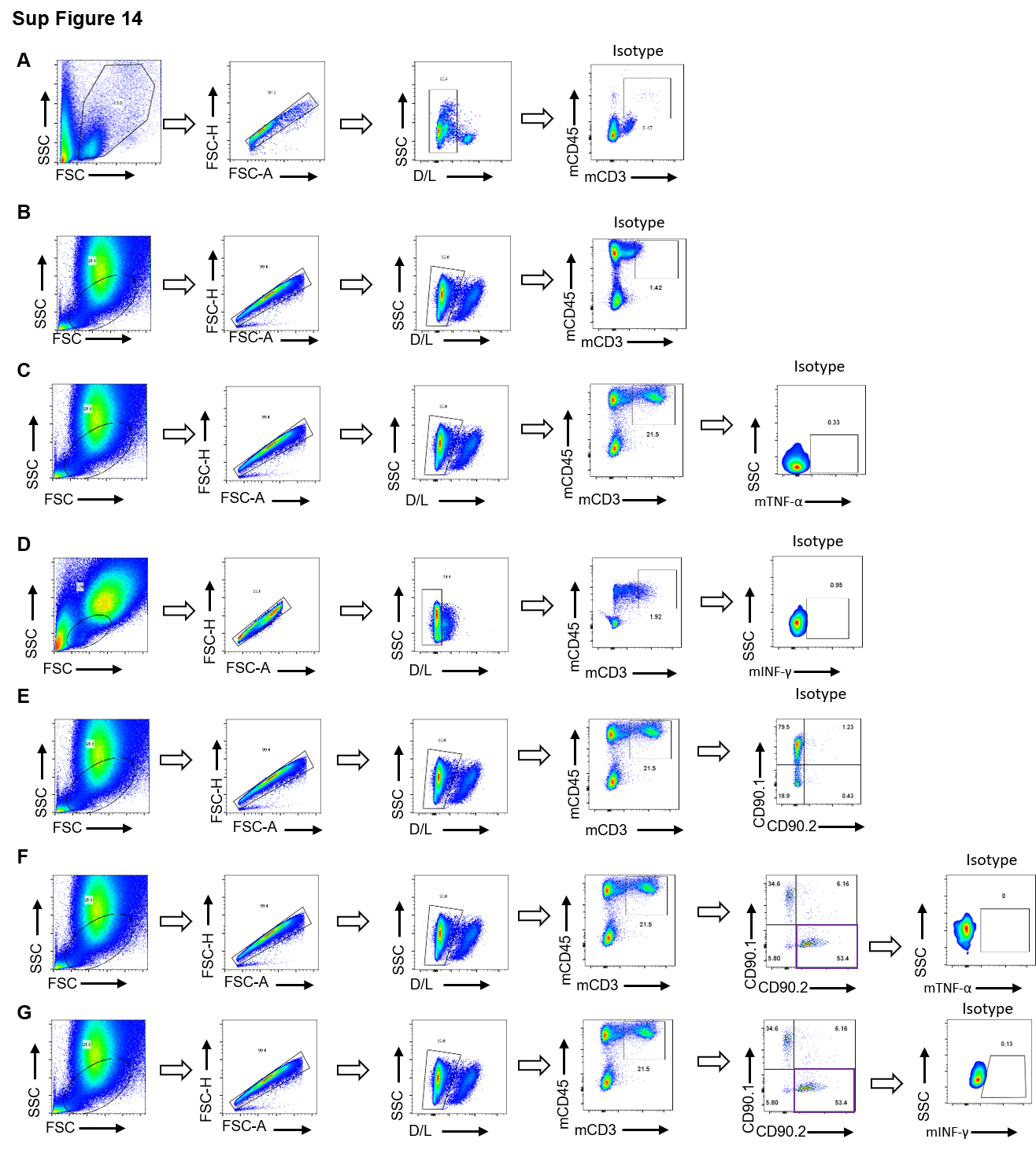


**Supplementary Figure S14. Gating strategies for adoptive T cells and cytokine analysis (related to Figure 6).** **A.** Total peripheral blood T cells (mCD3+). **B-D.** Total tumor‑infiltrating T cells: total (B), TNF‑α expression (C), and IFN‑γ expression (D). **E-G.** Tumor‑infiltrating CAR‑T cells (CD3+, CD90.2+): total (E), TNF‑α expression (F), and IFN‑γ expression (G).

**Supplementary Tables**

**Supplementary Table S1. Antibodies used for flow cytometry.**

| **Antibody name** | **Company** | | **clone** | **Catalog Number** |
| --- | --- | --- | --- | --- |
| PE anti-mCD3 | | BioLegend | 17A2 | 100206 |
| APC anti-mCD45 | BioLegend | | 30-F11 | 103112 |
| PE/Cy7 Anti-mCCL5 | BioLegend | | 2E9/CCL5 | 149105 |
| AF647 Anti-hEpCAM | BioLegend | | 9C4 | 324212 |
| AF647 anti-mouse Ab | BioLegend | | AF6-120.1 | 116412 |

**Supplementary Table S2. Antibodies used for Western blotting.**

| **Antibody name** | **Company** | **Catalog Number** | |
| --- | --- | --- | --- |
| myc | Cell Signaling Technology | | 2276 |
| phospho-JAK2 | Cell Signaling Technology | | 3771 |
| total-JAK2 | Cell Signaling Technology | | 3230 |
| phospho-PI3K | Cell Signaling Technology | | 17366 |
| total-PI3K | Cell Signaling Technology | | 4249 |
| phospho-Akt (Ser473) | Cell Signaling Technology | | 4060 |
| total-Akt | Cell Signaling Technology | | 2920 |
| phospho-ERK1/2 | Cell Signaling Technology | | 4370 |
| total- ERK1/2 | Cell Signaling Technology | | 4695 |
| phospho-STAT3 | Cell Signaling Technology | | 12640 |
| total- STAT3 | Cell Signaling Technology | | 9145 |
| phospho-JNK | Cell Signaling Technology | | 4668 |
| total-JNK | Cell Signaling Technology | | 9252 |
| phospho-NF-κB (p65) | Cell Signaling Technology | | 3033 |
| total- NF-κB (p65) | Cell Signaling Technology | | 8242 |
| phospho-IκBα | Cell Signaling Technology | | 2859 |
| total-IκBα | Cell Signaling Technology | | 9242 |
| CCR5 | Santa Cruz | | sc-32304 |
| GAPDH | Proteintech | | 60004-1-Ig |

**Supplementary Table S3. Primer sequences used for qPCR.**

| Genes | Forward primer | Reverse primer |
| --- | --- | --- |
| *Ccl5* | CCAGCAGTCGTCTTTGTCAC | CTCTGGGTTGGCACACACTT |
| *Vegf* | AGGGCAGAATCATCACGAAGT | AGGGTCTCGATTGGATGGCA |
| *Gapdh* | GGAGCGAGATCCCTCCAAAAT | GGCTGTTGTCATACTTCTCATGG |

**Supplementary Table S4. The characteristics of the patients enrolled in clinic trials.**

| ID | Primary Lesion | Stage grouping | Prior Chemotherapy | CAR-T dose (cells/kg) |
| --- | --- | --- | --- | --- |
| 1 | Stomach | IV | Oxaliplatin, Tislelizumab | 6×10^6 |
| 2 | Ovary | Ia | Paclitaxel, Carboplatin,Bevacizumab | 1×10^6 |
| 3 | Prostate | IV | Enzalutamide, Bicalutamide, Goserelin, Abiraterone, Prednisolone | 1×10^6 |
| 4 | Prostate | IV | Abiraterone, Prednisone, Goserelin, Relugolix | 1×10^6 |
| 5 | Nasopharynx | IV | Tegafur, Nedaplatin, S-1, Capecitabine, Camrelizumab, Toripalimab | 3×10^6 |
